# Supplementary material for: Confined migration promotes cancer metastasis through resistance to anoikis and increased invasiveness
Source: eLife. 2022 Mar 8;11:e73150. doi: 10.7554/eLife.73150 (PMC8903834; doi:10.7554/eLife.73150)
Supplement: Source data 1. [file elife-73150-data1.zip › 2022 Fanfone et al. source data WB panels/Fig2H source data ANNOTATED.pdf]

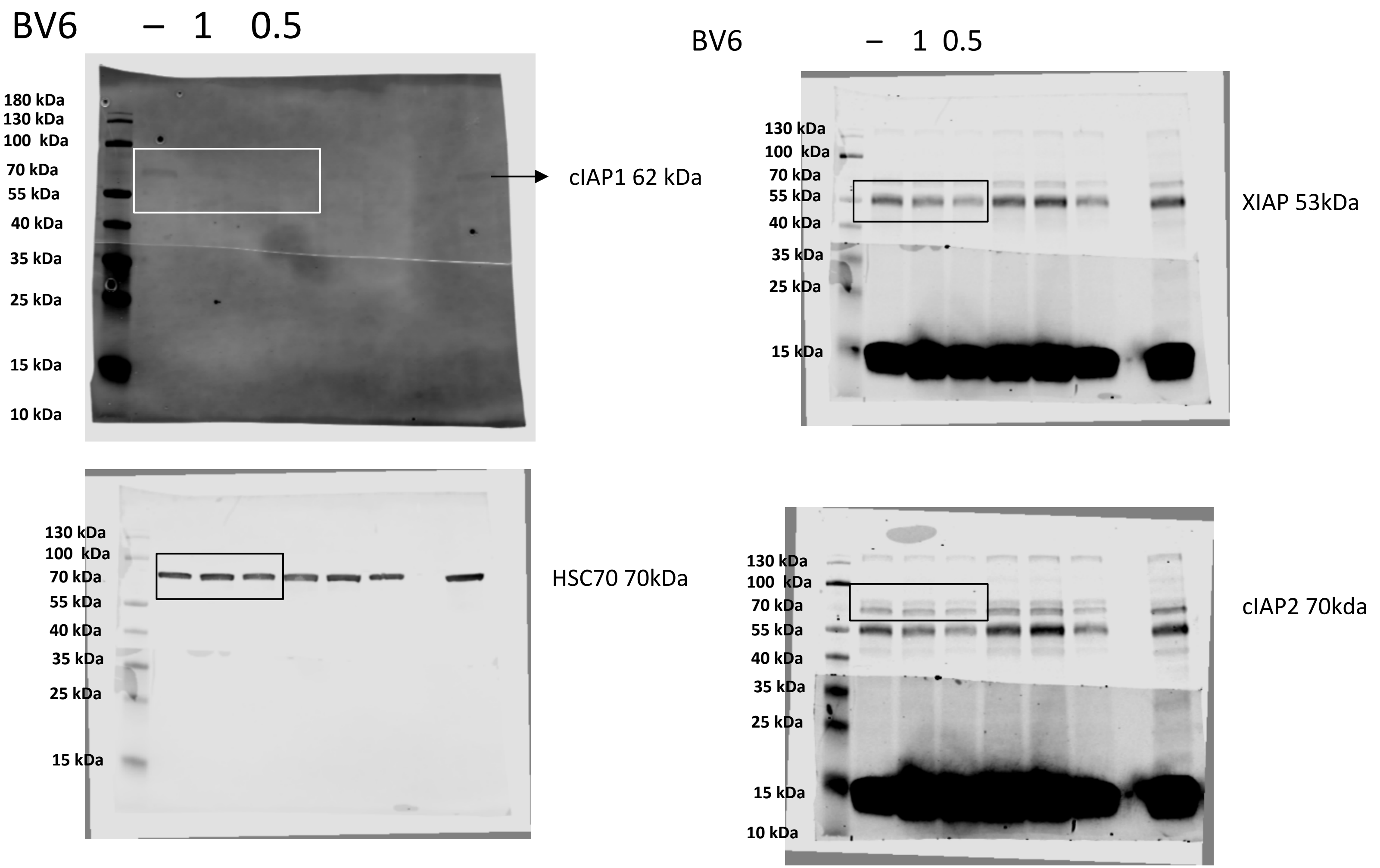
BV6 – 1 0.5
180 kDa
130 kDa
100 kDa
70 kDa
55 kDa
40 kDa
35 kDa
25 kDa
15 kDa
10 kDa
cIAP1 62 kDa
BV6 – 1 0.5
130 kDa
100 kDa
70 kDa
55 kDa
40 kDa
35 kDa
25 kDa
15 kDa
XIAP 53kDa
130 kDa
100 kDa
70 kDa
55 kDa
40 kDa
35 kDa
25 kDa
15 kDa
HSC70 70kDa
130 kDa
100 kDa
70 kDa
55 kDa
40 kDa
35 kDa
25 kDa
15 kDa
10 kDa
cIAP2 70kda

Figure 2H
