# Supplementary figures and images for: Confined migration promotes cancer metastasis through resistance to anoikis and increased invasiveness
Source: eLife. 2022 Mar 8;11:e73150. doi: 10.7554/eLife.73150 (PMC8903834; doi:10.7554/eLife.73150)

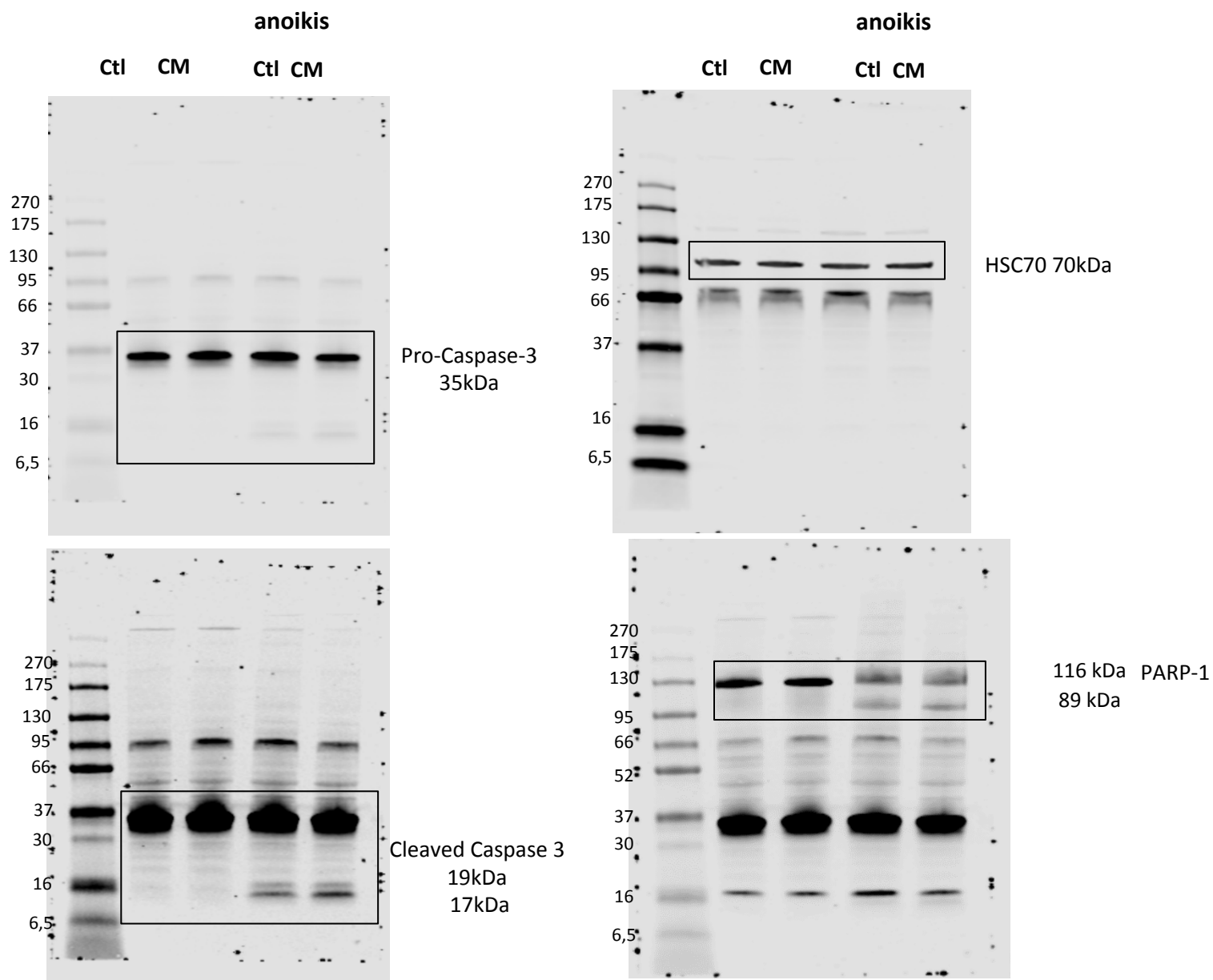

Supplementary Fig. 2B

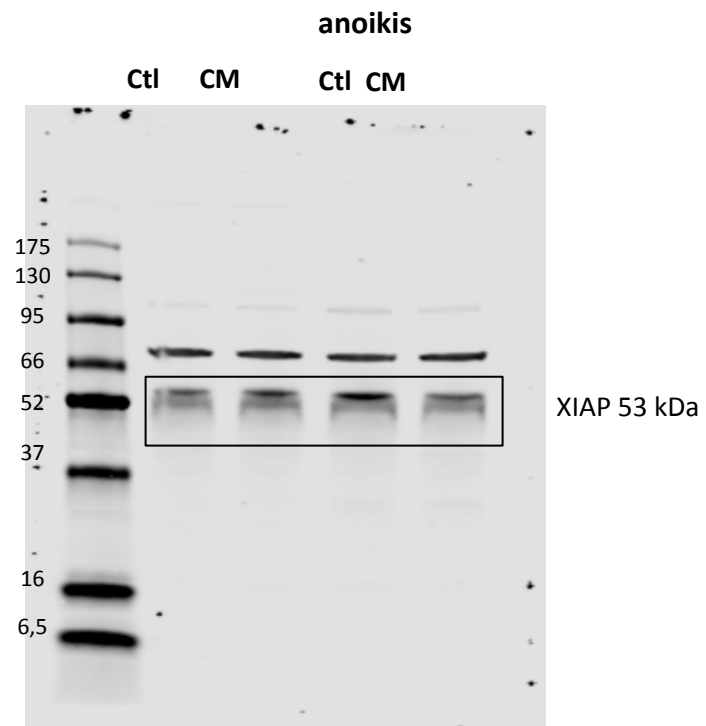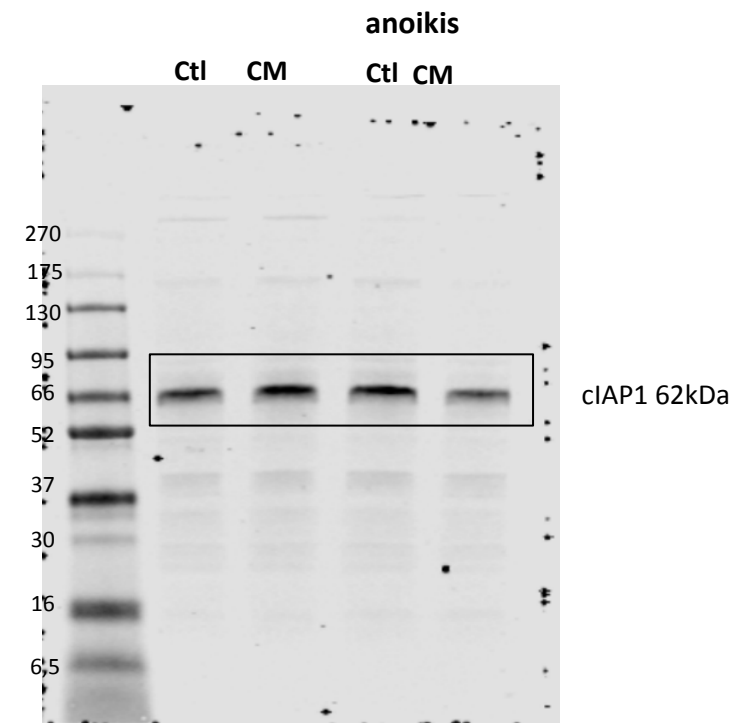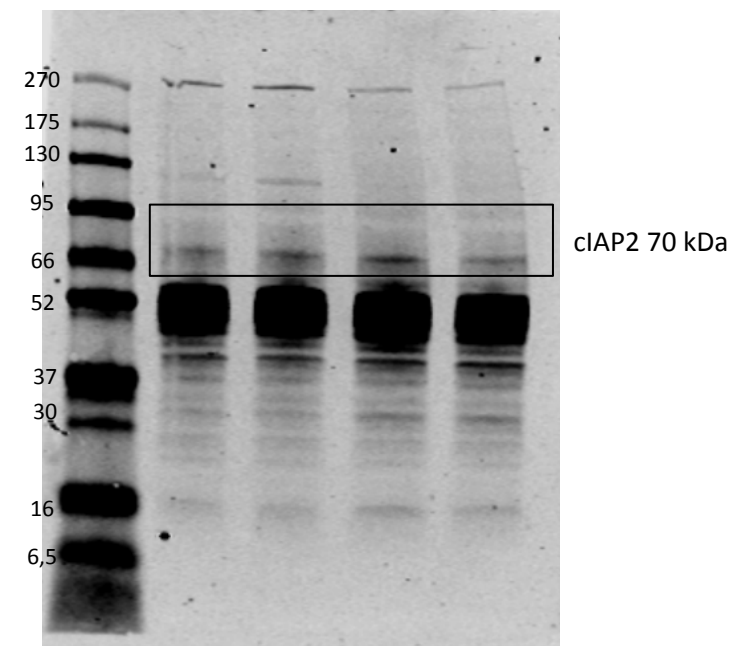

Supplementary Fig. 2B

Supplement: Source data 1. [file elife-73150-data1.zip › 2022 Fanfone et al. source data WB panels/FigS2B source data ANNOTATED.pdf]

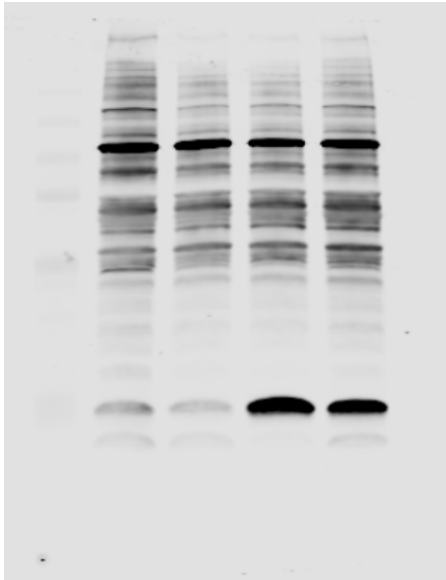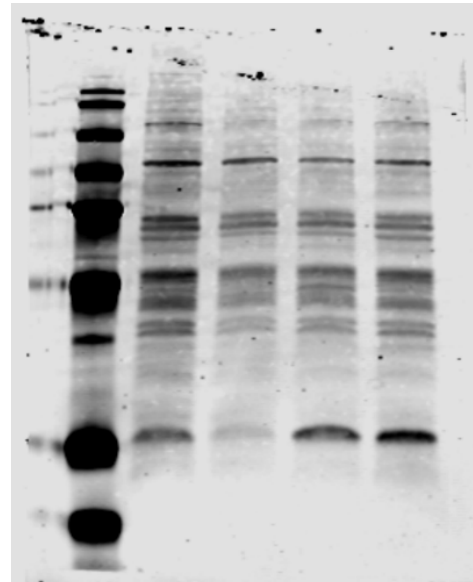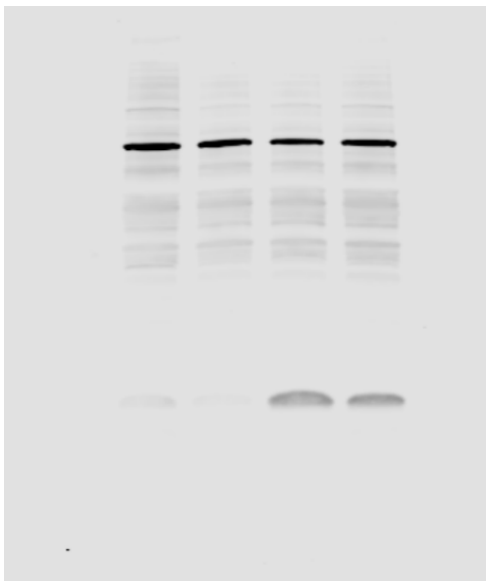

Supplementary Fig. 3C

Supplement: Source data 1. [file elife-73150-data1.zip › 2022 Fanfone et al. source data WB panels/FigS3C source data RAW.pdf]

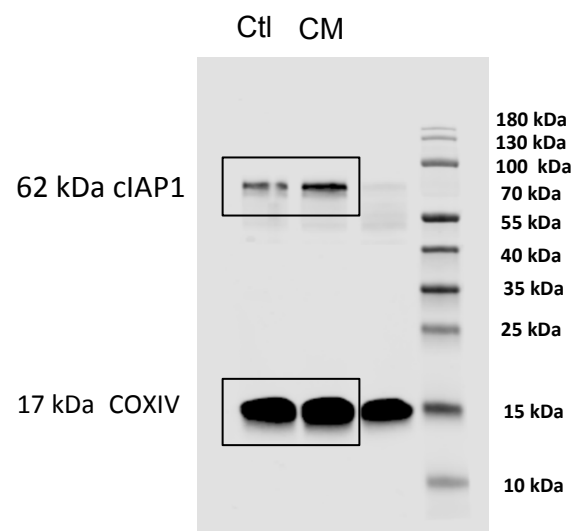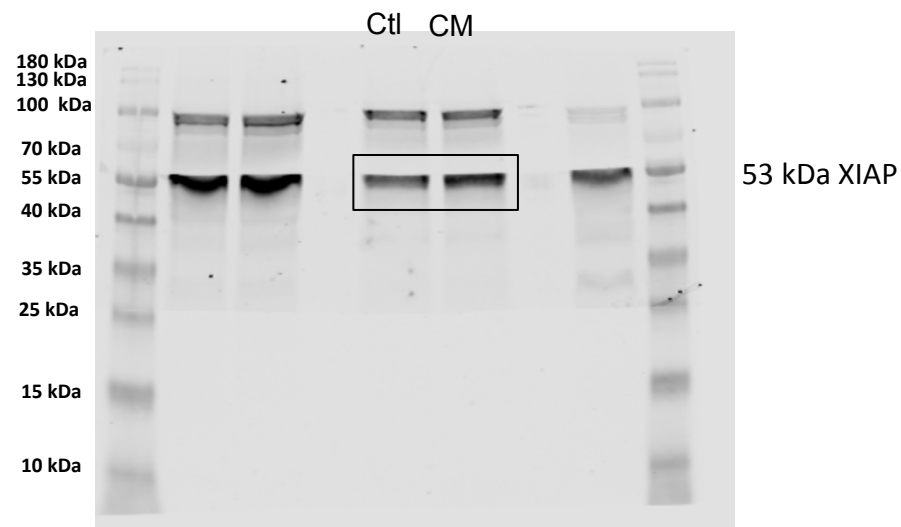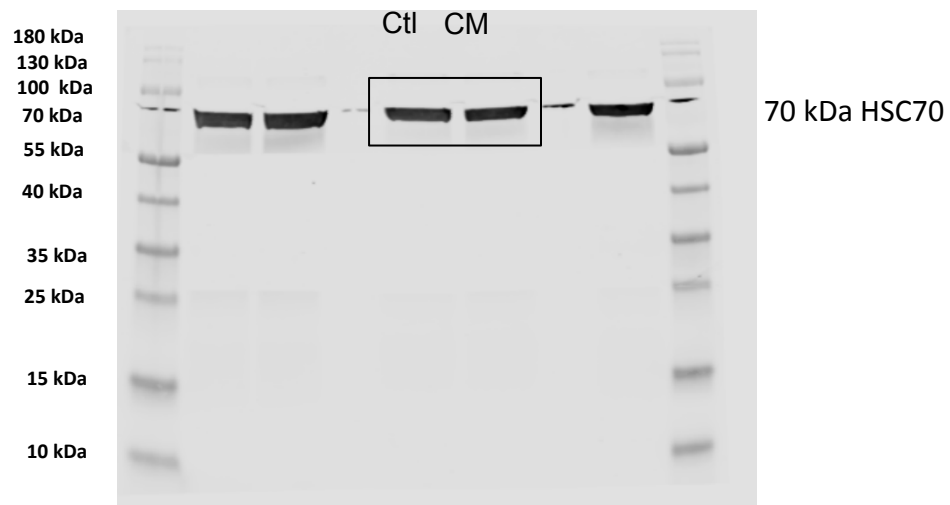

Figure 2A

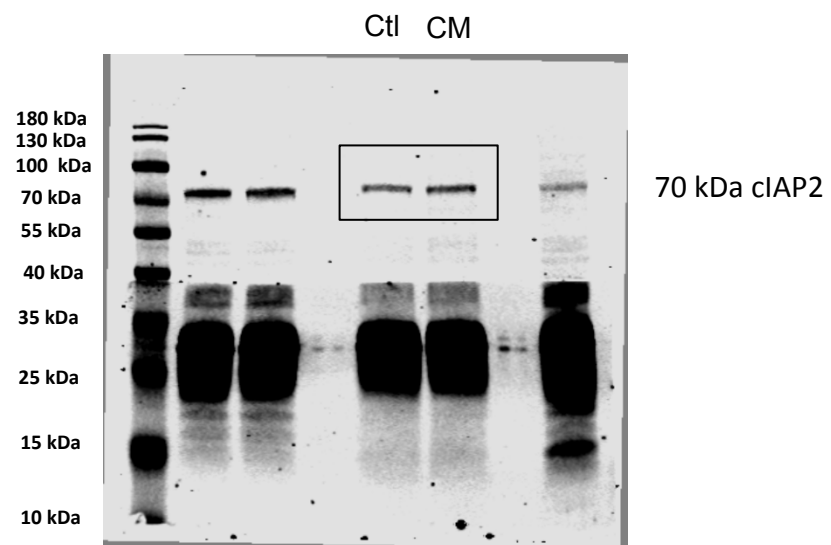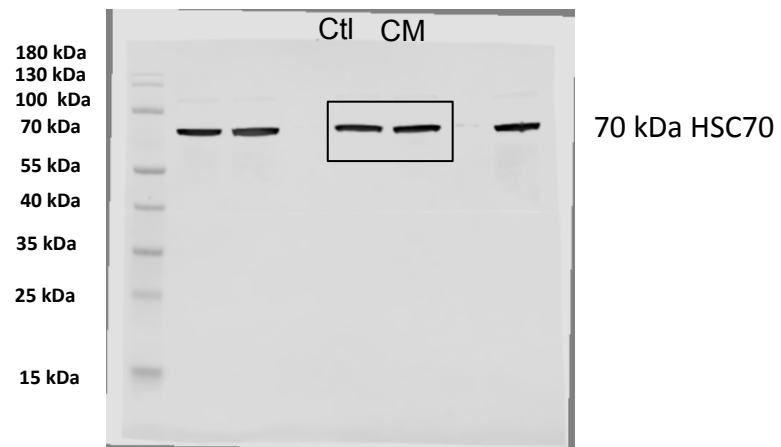

Figure 2A

Supplement: Source data 1. [file elife-73150-data1.zip › 2022 Fanfone et al. source data WB panels/Fig2A source data ANNOTATED.pdf]

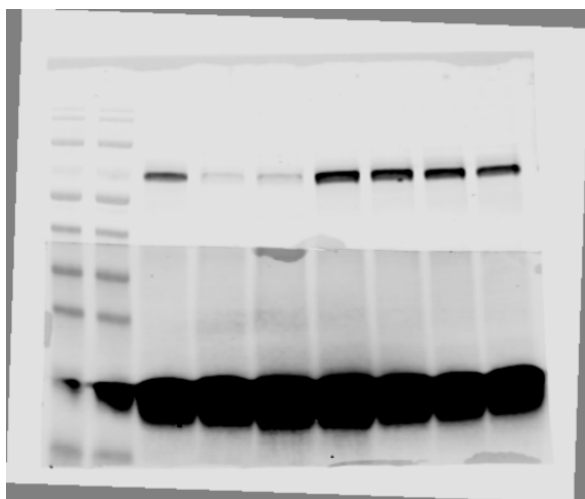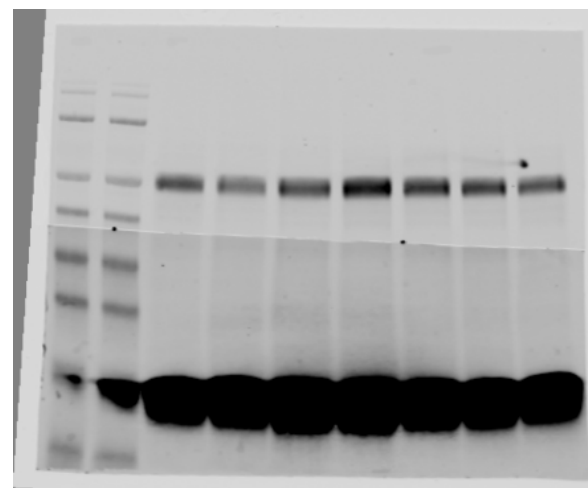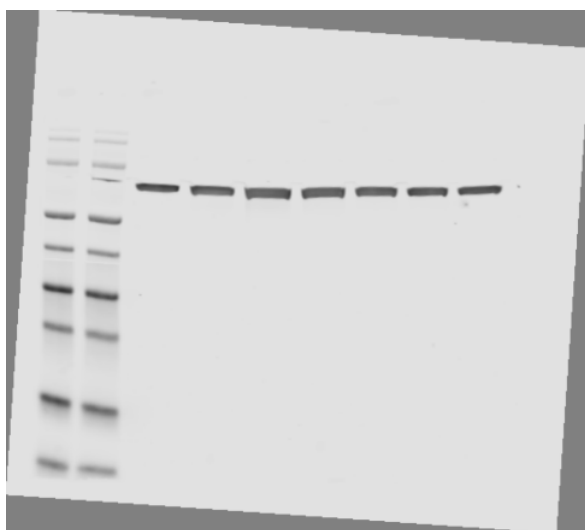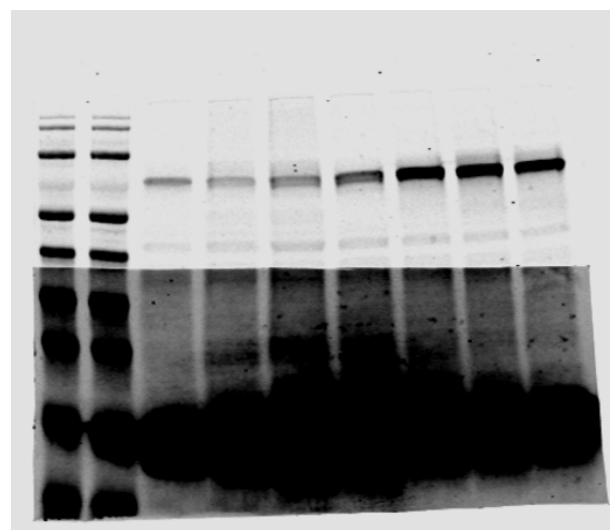

Supplementary Fig. 2N

Supplement: Source data 1. [file elife-73150-data1.zip › 2022 Fanfone et al. source data WB panels/FigS2N source data RAW.pdf]

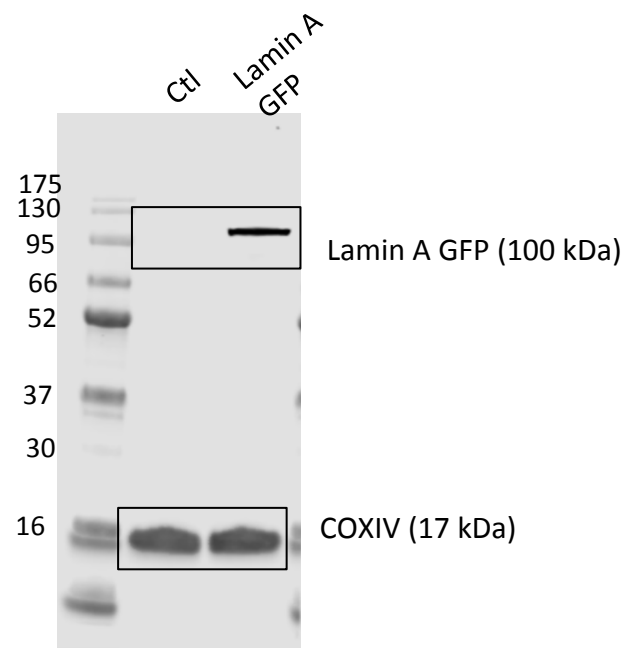

Supplementary Fig. 3H

Supplement: Source data 1. [file elife-73150-data1.zip › 2022 Fanfone et al. source data WB panels/FigS3H source data ANNOTATED.pdf]

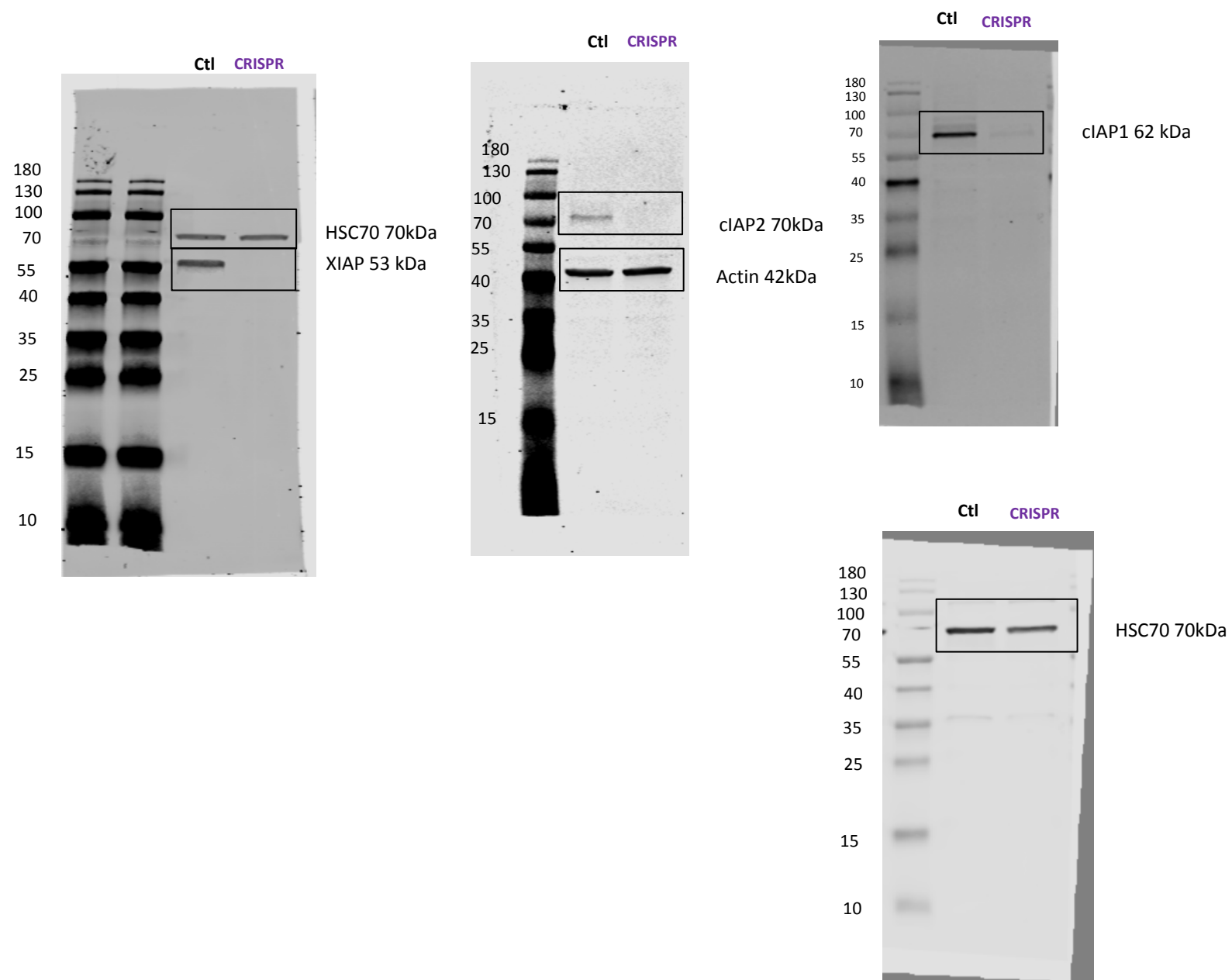

Figure 2F

Supplement: Source data 1. [file elife-73150-data1.zip › 2022 Fanfone et al. source data WB panels/Fig2F source data ANNOTATED.pdf]

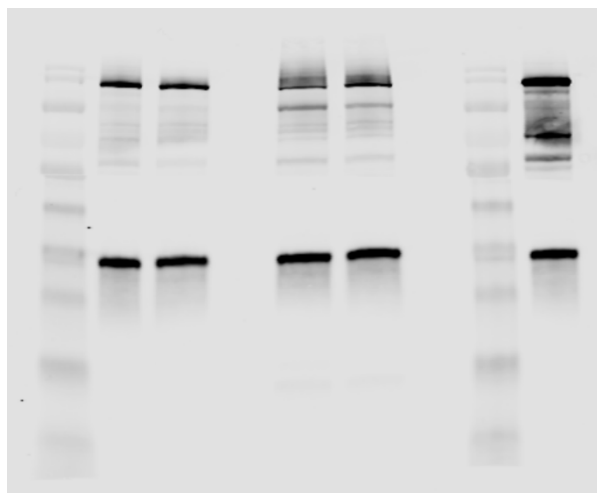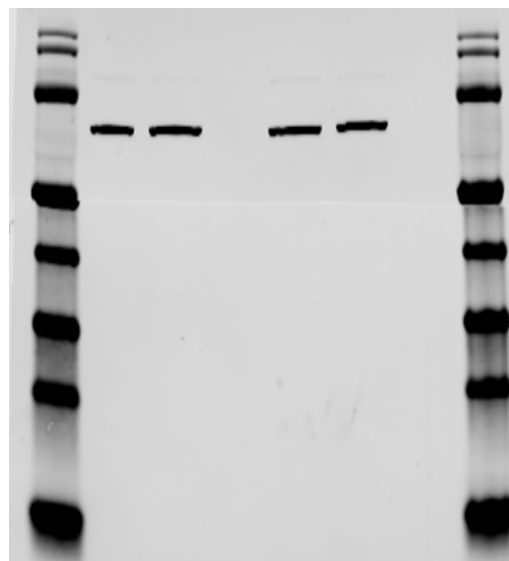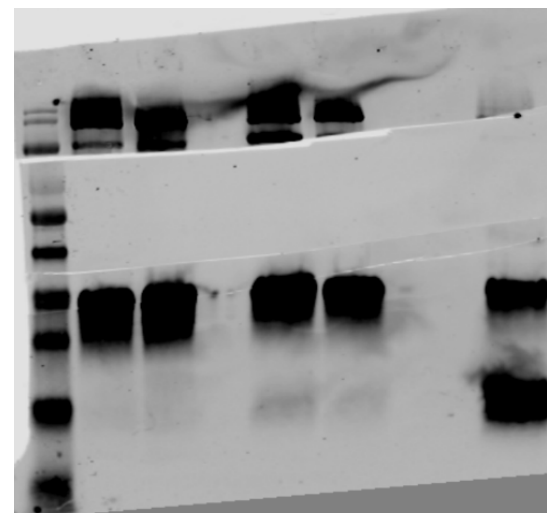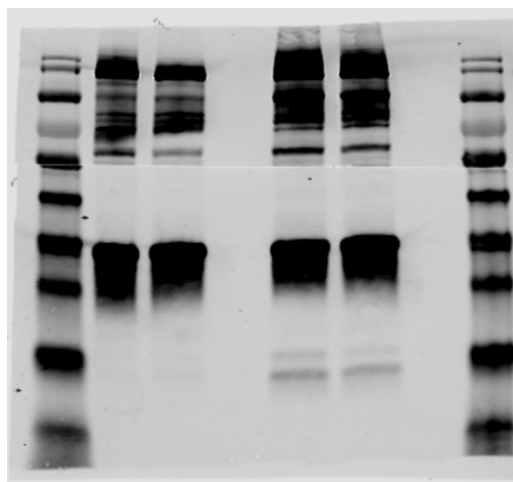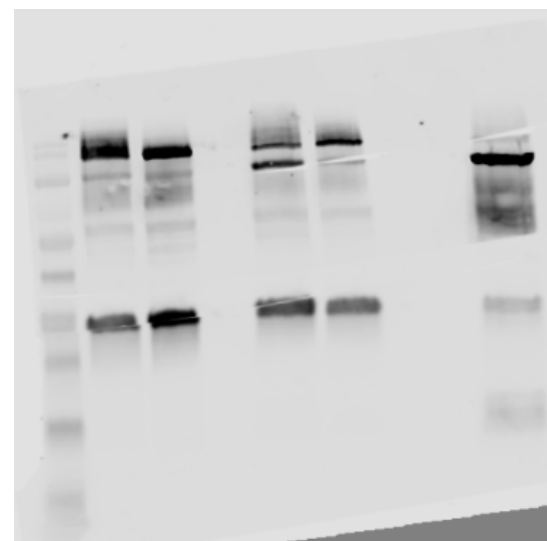

CRISPR<sup>Ctl</sup> MDA-MB-231

Figure 1K

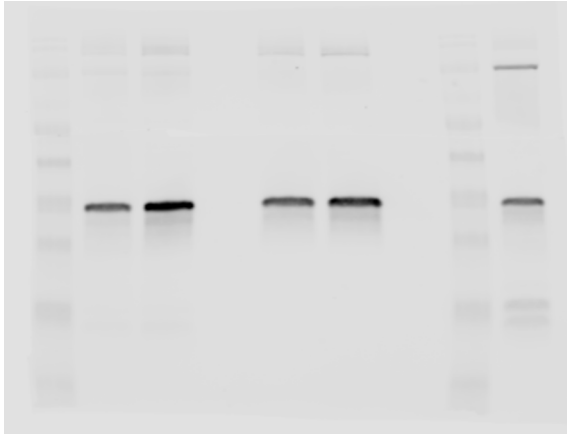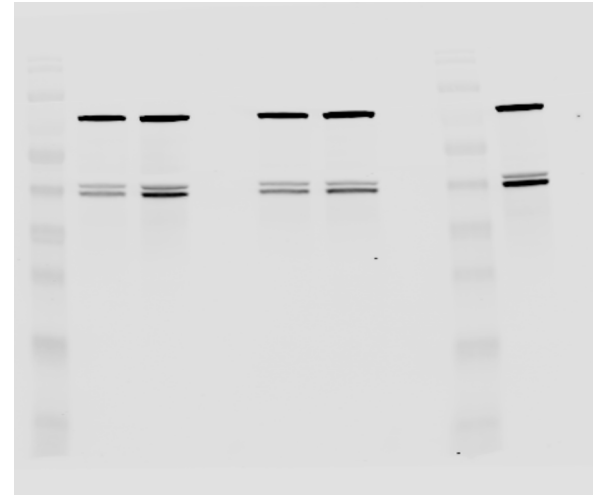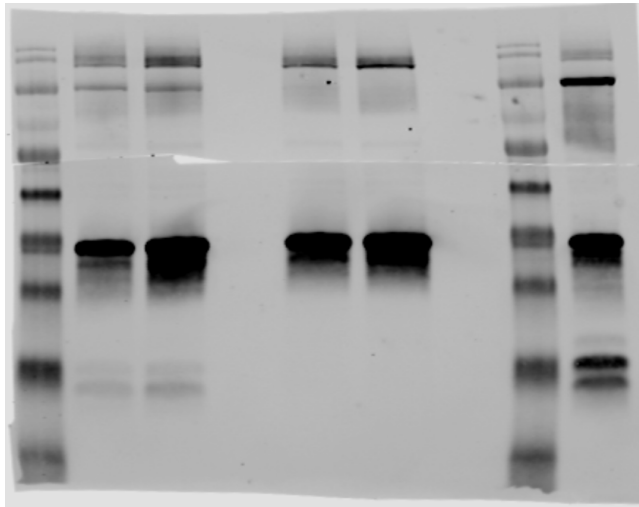

Figure 1K

CRISPR <sup>BAX/BAK</sup> MDA-MB-231

Supplement: Source data 1. [file elife-73150-data1.zip › 2022 Fanfone et al. source data WB panels/Fig1k source data RAW.pdf]

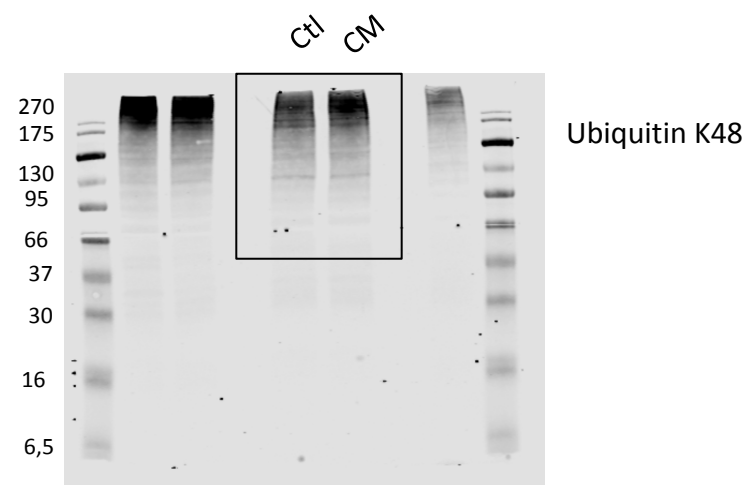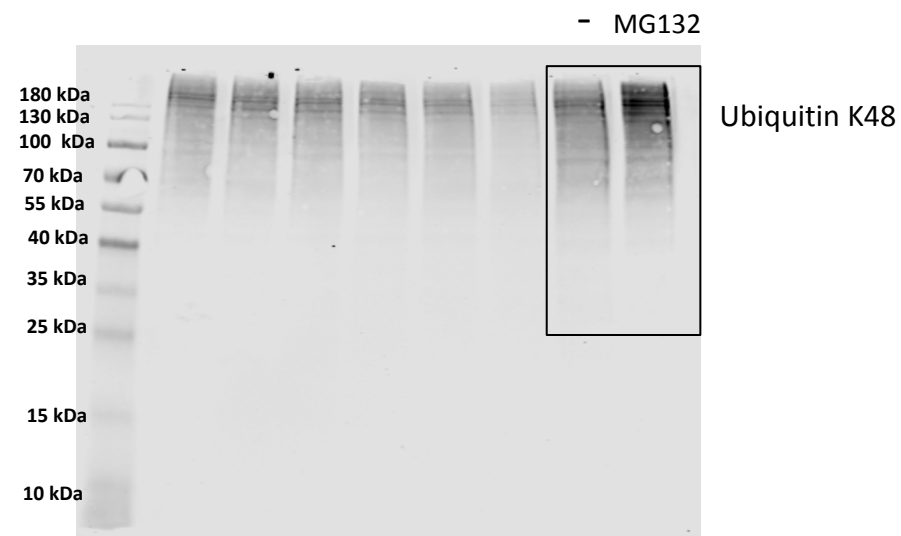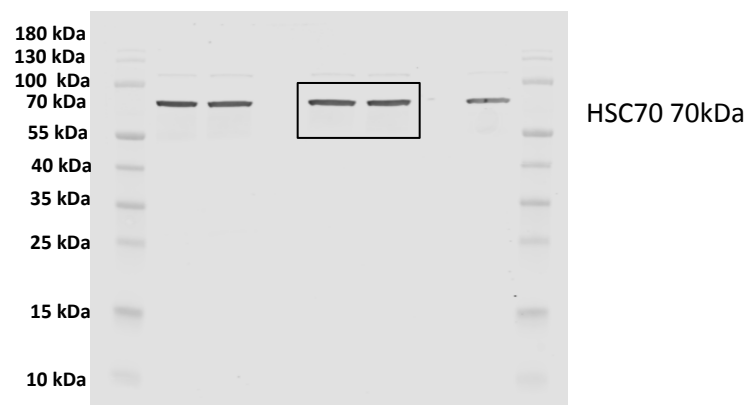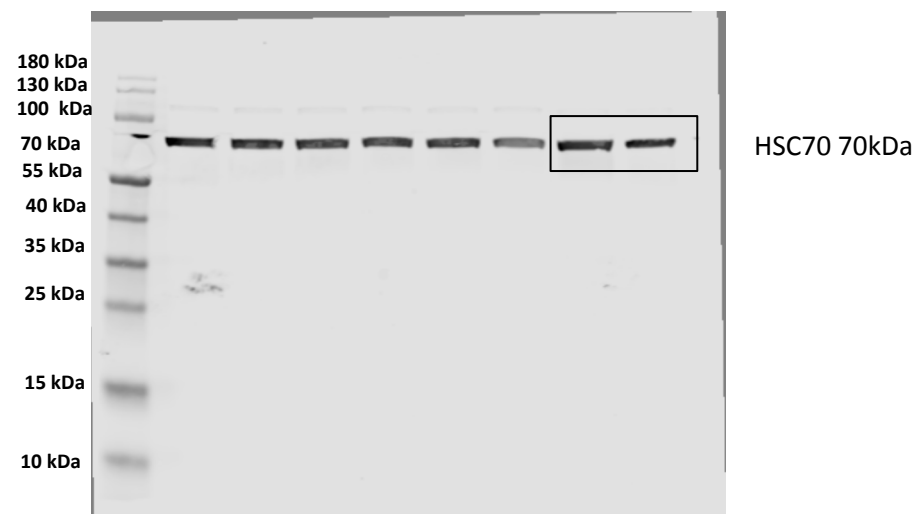

Supplementary Fig. 2E

Supplement: Source data 1. [file elife-73150-data1.zip › 2022 Fanfone et al. source data WB panels/FigS2E source data ANNOTATED.pdf]

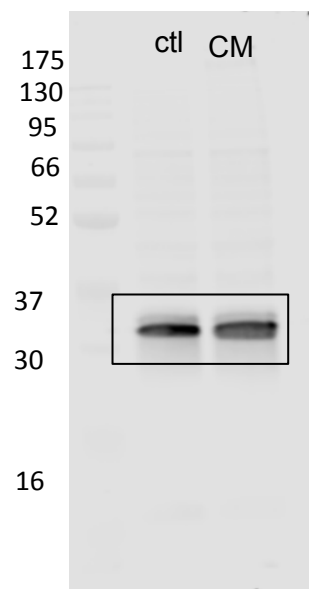

BCLxL (30 kDa)

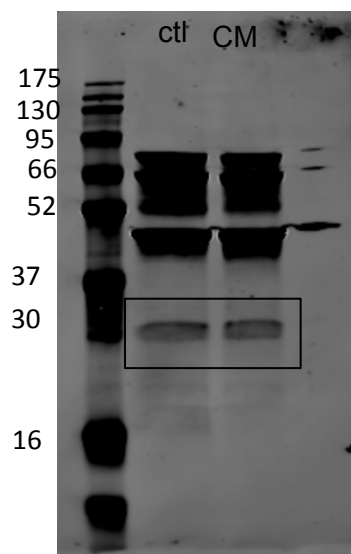

BCL2 (26 kDa)

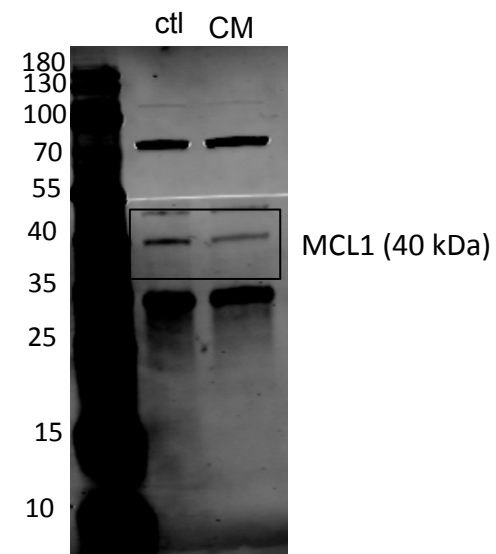

MCL1 (40 kDa)

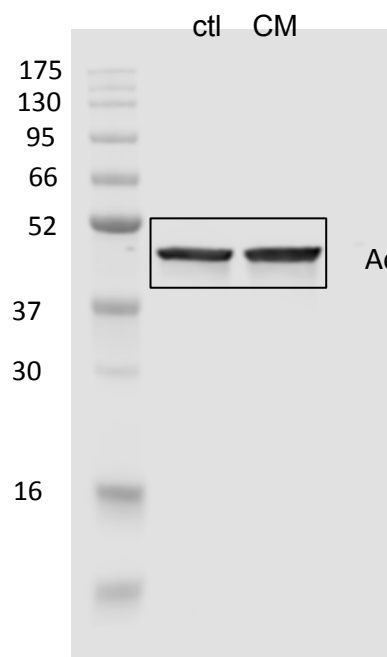

Actin (42 kDa)

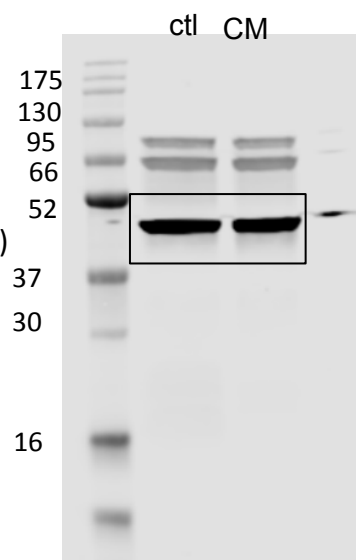

Actin (42 kDa)

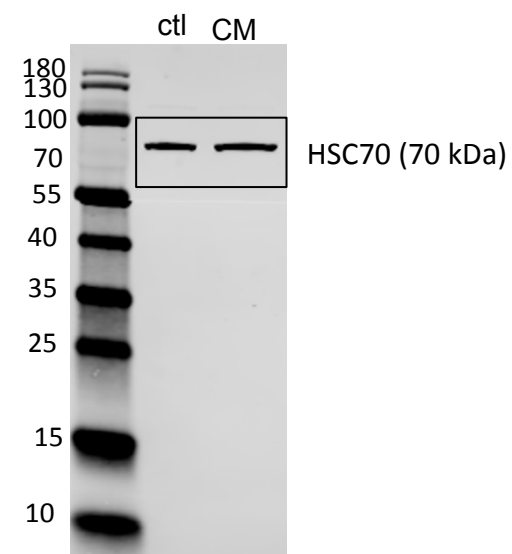

HSC70 (70 kDa)

Supplementary Figure 1P

Supplement: Source data 1. [file elife-73150-data1.zip › 2022 Fanfone et al. source data WB panels/FigS1P source data ANNOTATED.pdf]

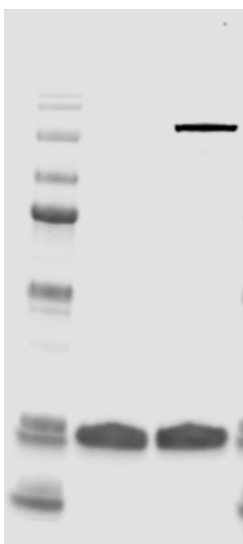

Supplementary Fig. 3H

Supplement: Source data 1. [file elife-73150-data1.zip › 2022 Fanfone et al. source data WB panels/FigS3H source data RAW.pdf]

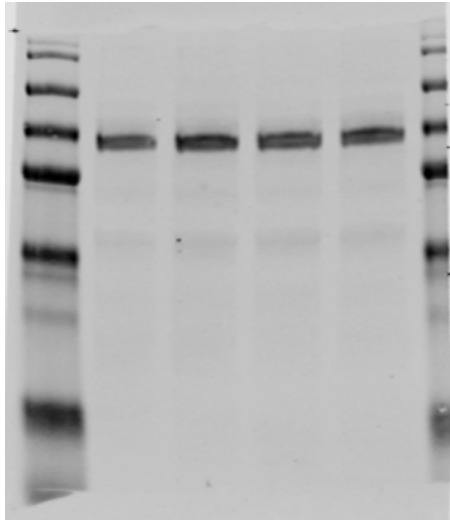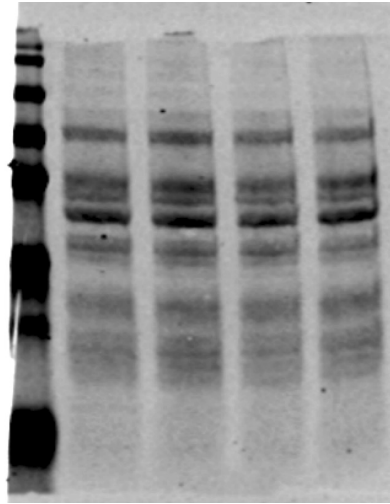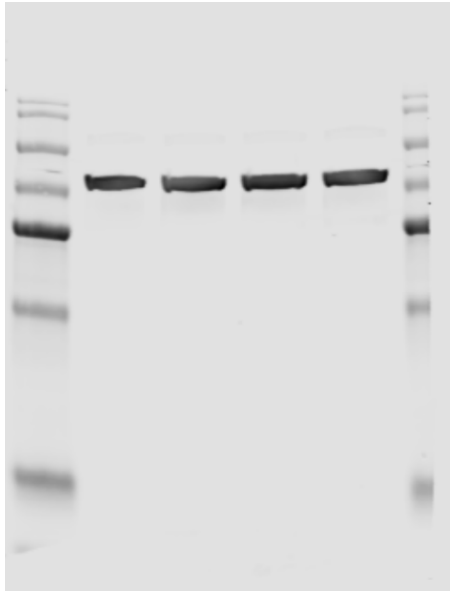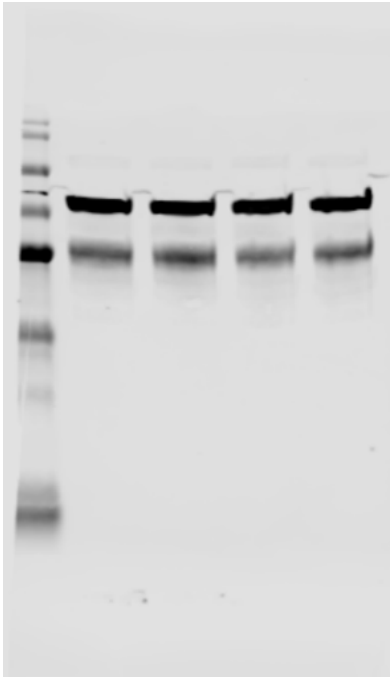

Supplementary Fig. 2A

Supplement: Source data 1. [file elife-73150-data1.zip › 2022 Fanfone et al. source data WB panels/FigS2A source data RAW.pdf]

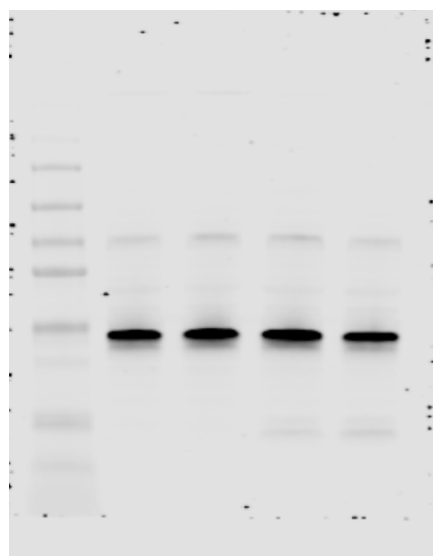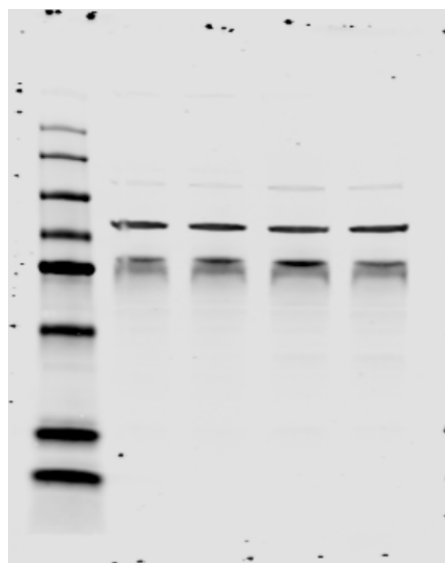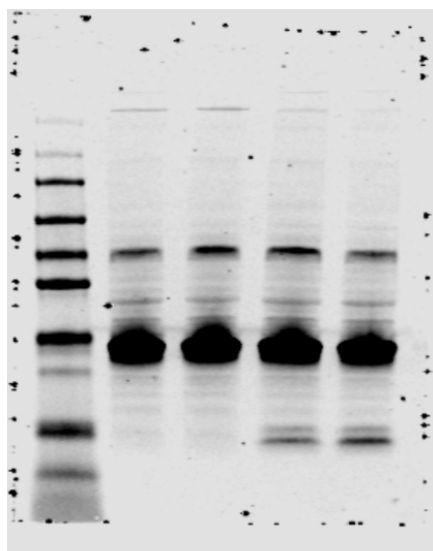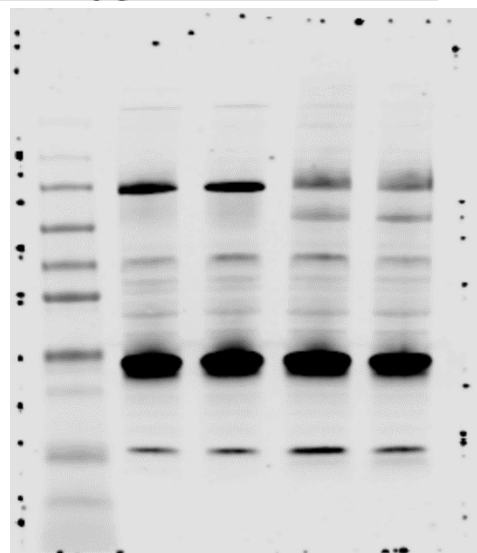

Supplementary Fig. 2B

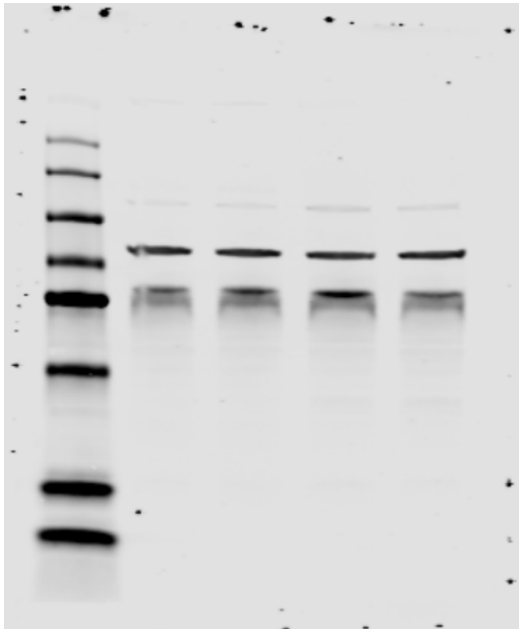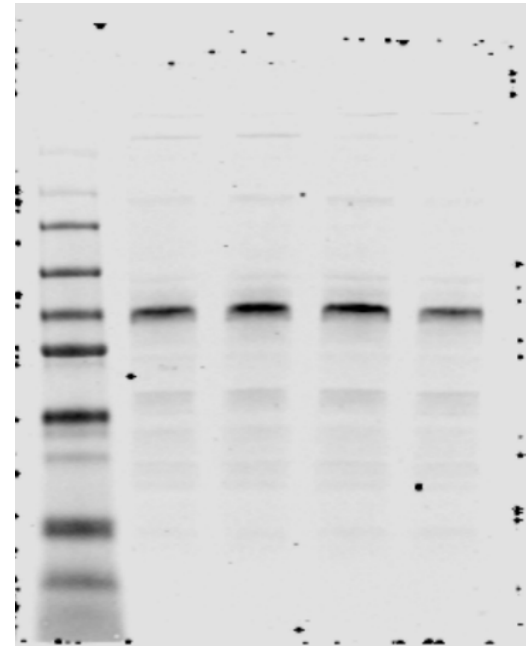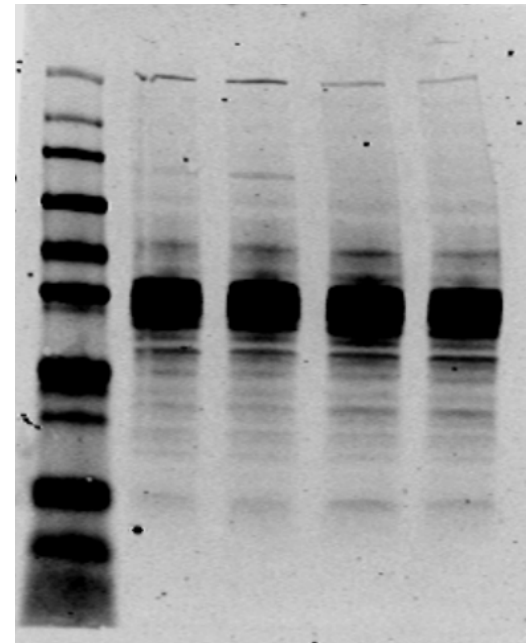

Supplementary Fig. 2B

Supplement: Source data 1. [file elife-73150-data1.zip › 2022 Fanfone et al. source data WB panels/FigS2B source data RAW.pdf]

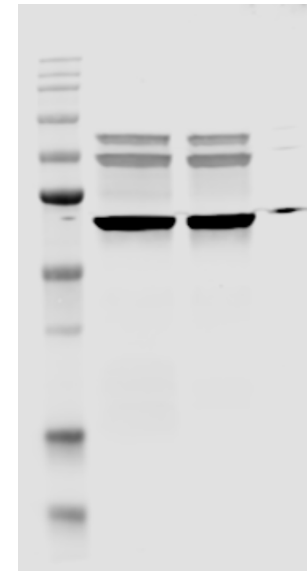

Supplementary Fig. 3K

Supplement: Source data 1. [file elife-73150-data1.zip › 2022 Fanfone et al. source data WB panels/FigS3K source data RAW.pdf]

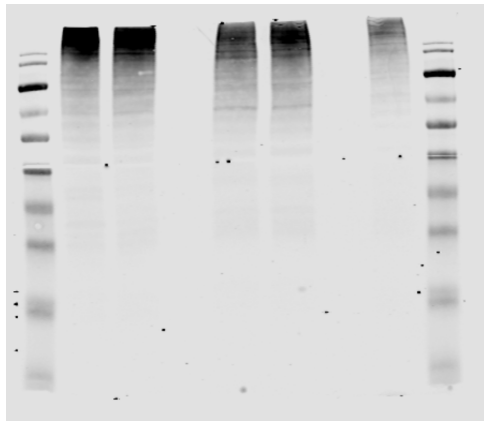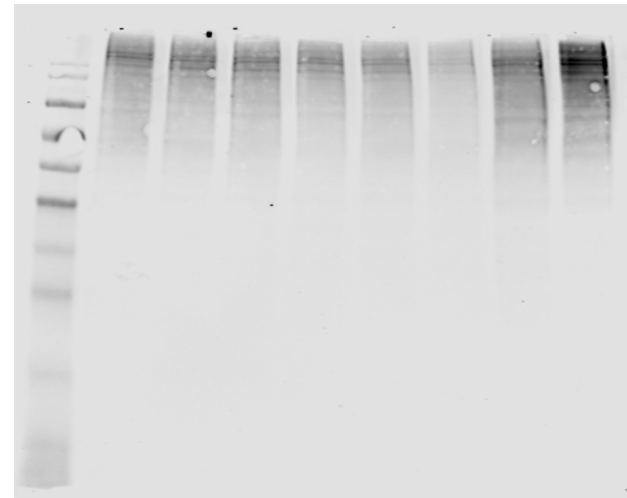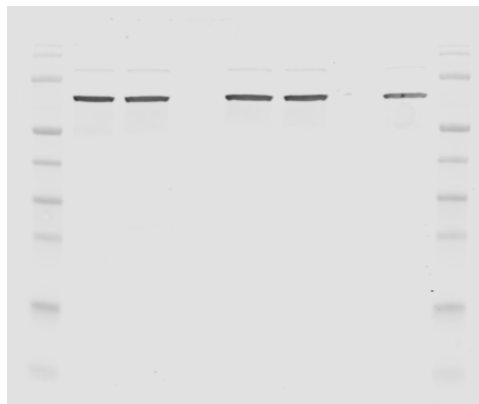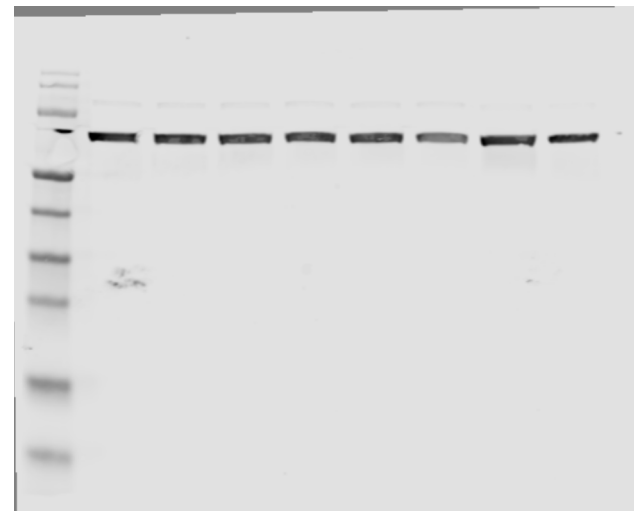

Supplementary Fig. 2E

Supplement: Source data 1. [file elife-73150-data1.zip › 2022 Fanfone et al. source data WB panels/FigS2E source data RAW.pdf]

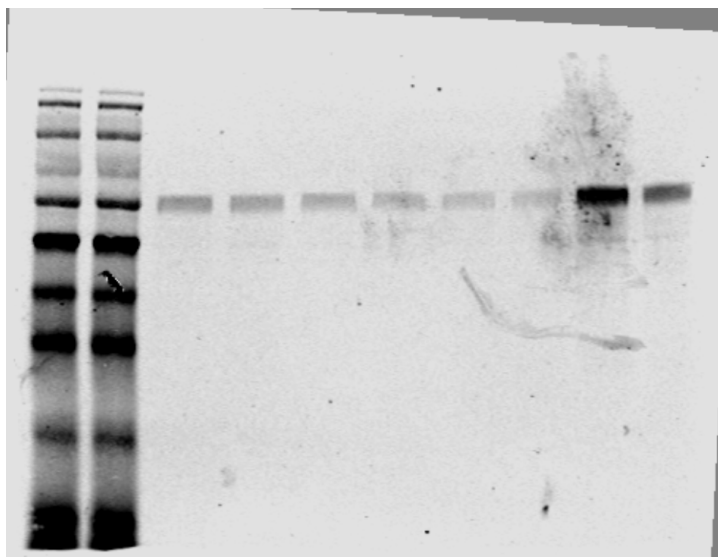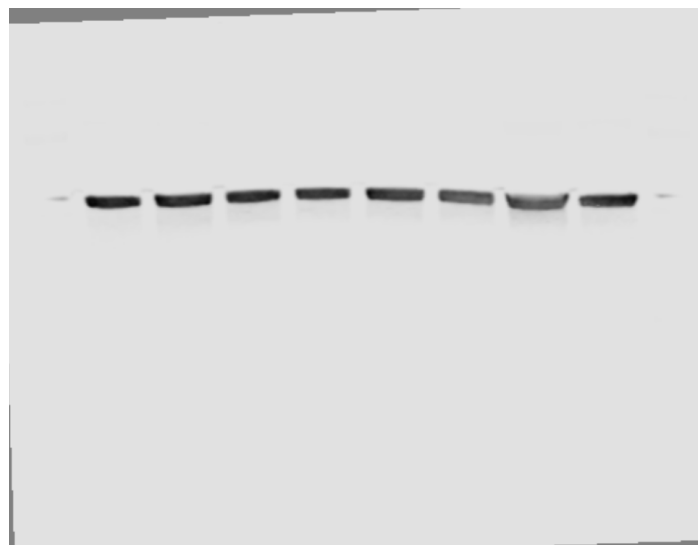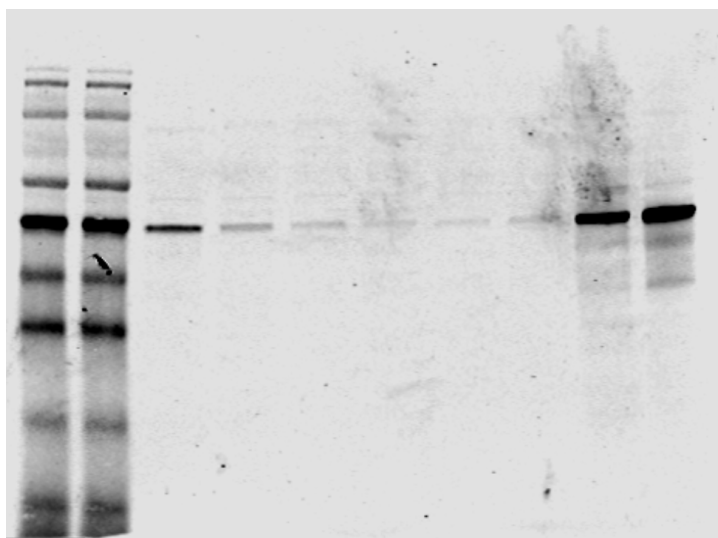

Supplementary Fig. 2F

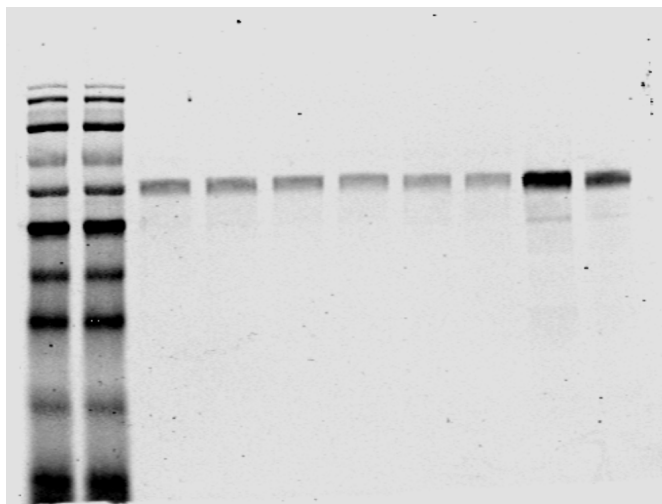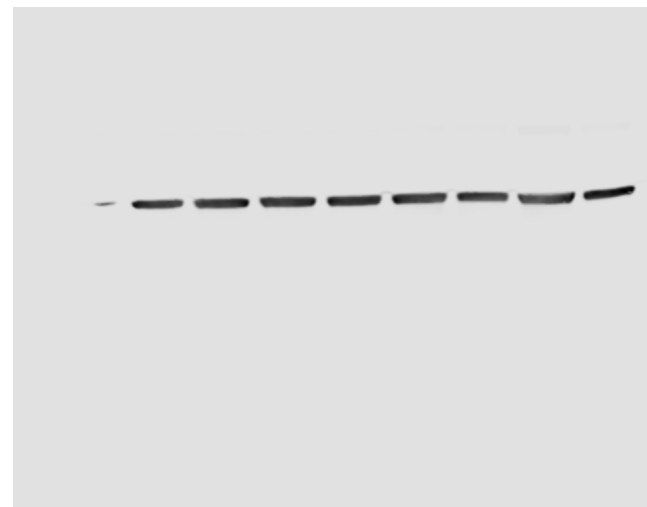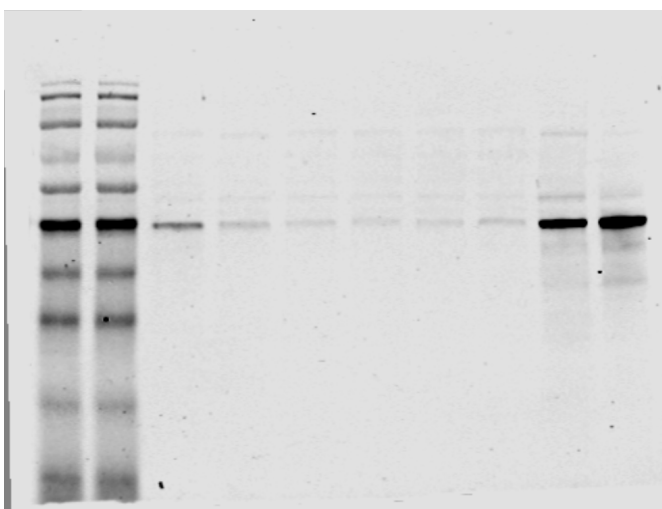

Supplementary Fig. 2F

Supplement: Source data 1. [file elife-73150-data1.zip › 2022 Fanfone et al. source data WB panels/FigS2F source data RAW.pdf]

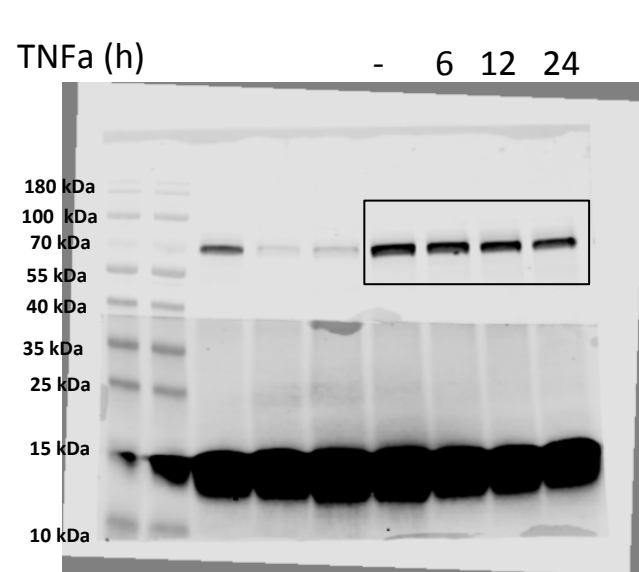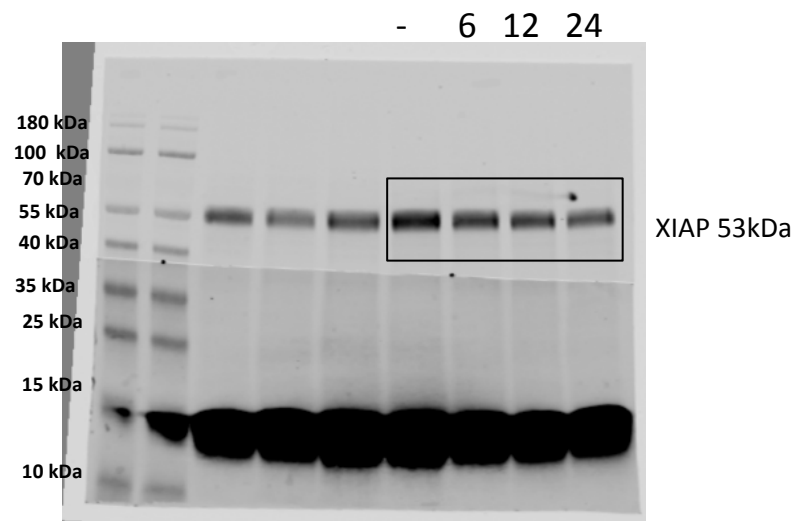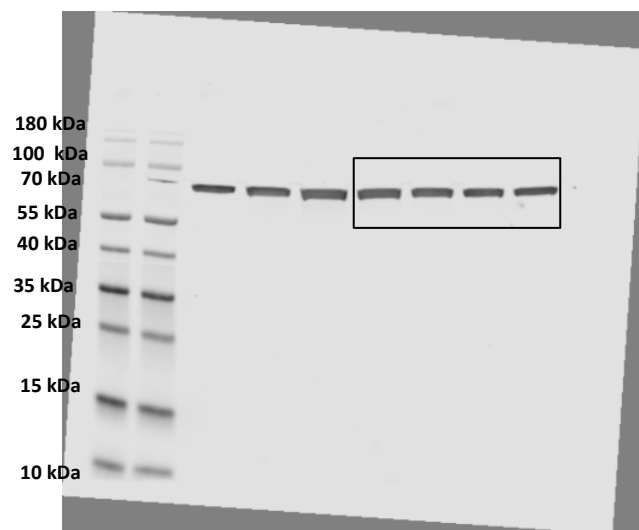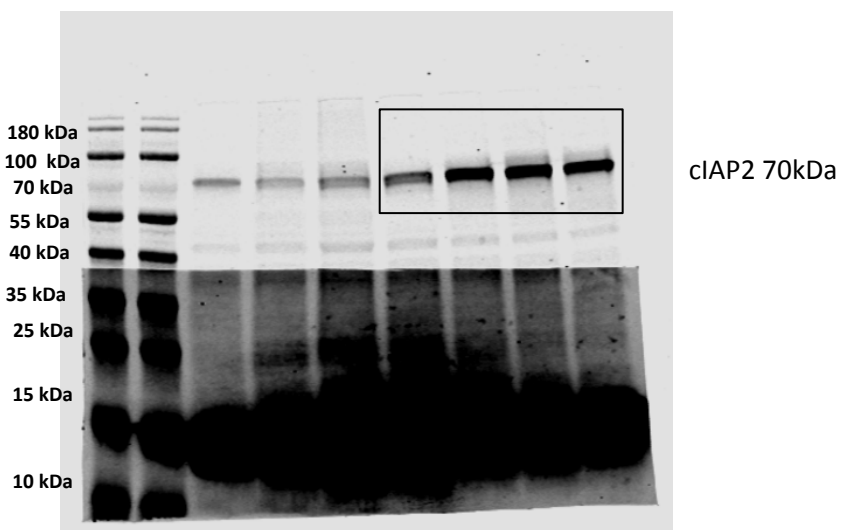

Supplementary Fig. 2N

Supplement: Source data 1. [file elife-73150-data1.zip › 2022 Fanfone et al. source data WB panels/FigS2N source data ANNOTATED.pdf]

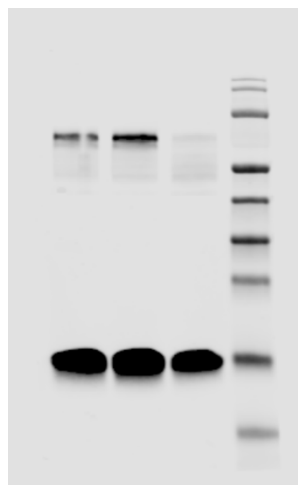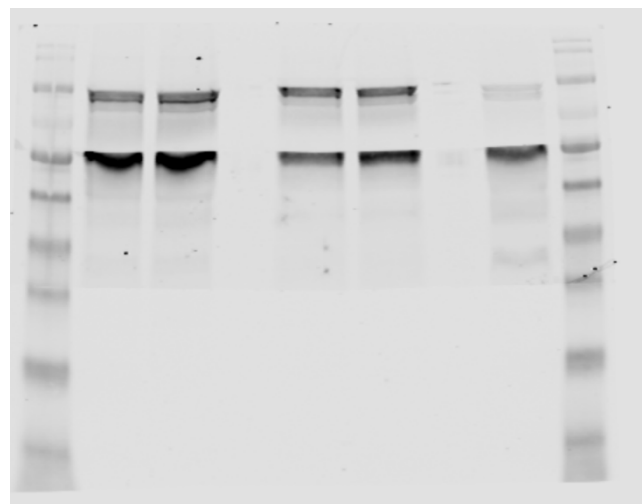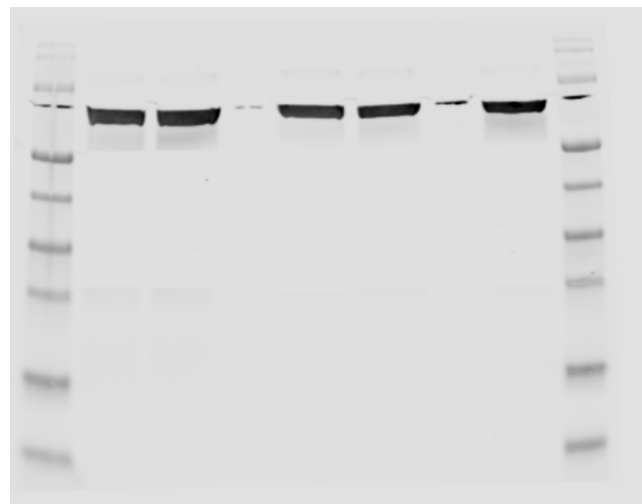

Figure 2A

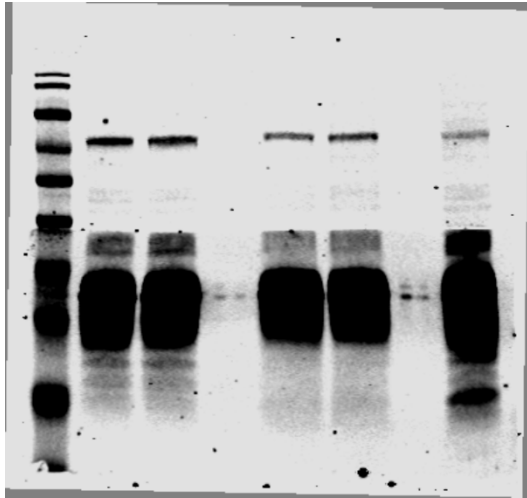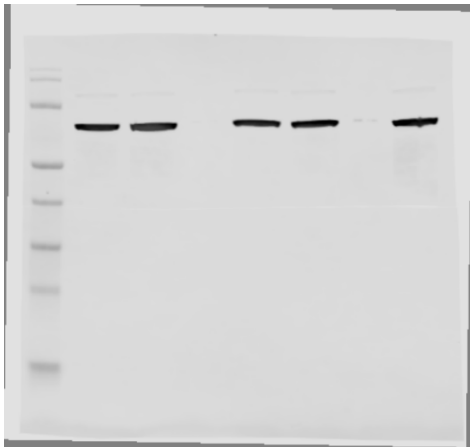

Figure 2A

Supplement: Source data 1. [file elife-73150-data1.zip › 2022 Fanfone et al. source data WB panels/Fig2A source data RAW.pdf]

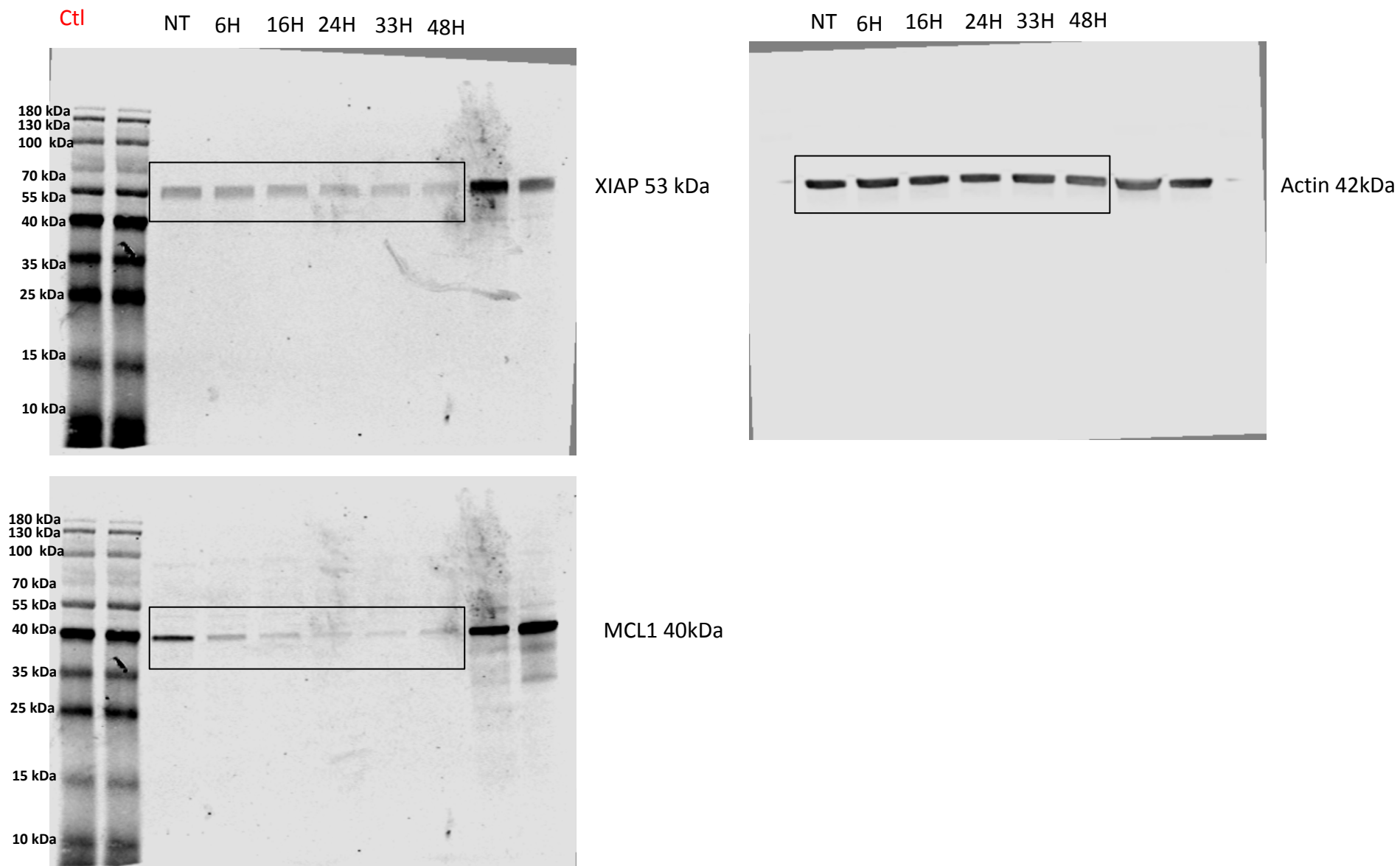

Supplementary Fig. 2F

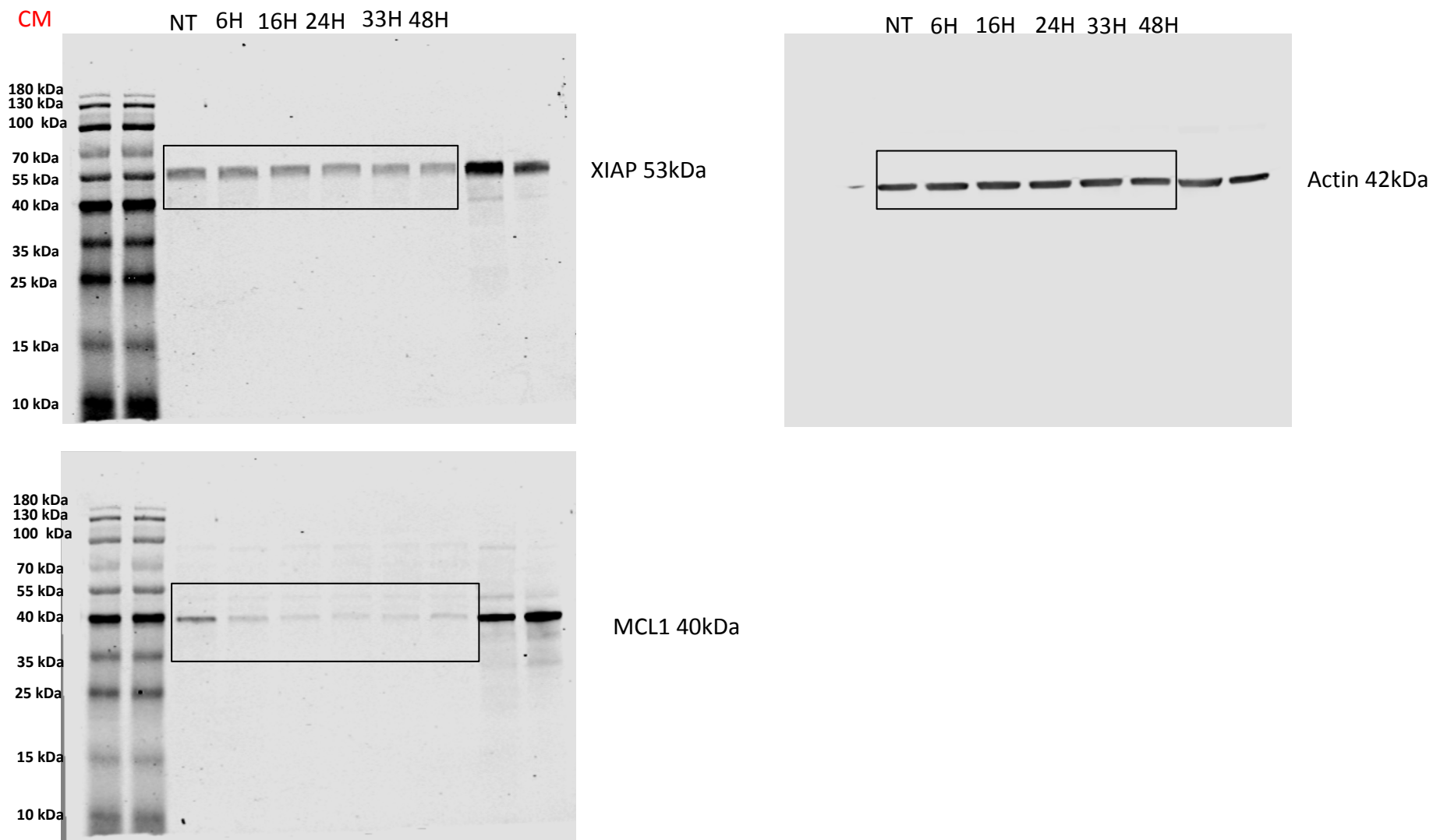

Supplementary Fig. 2F

Supplement: Source data 1. [file elife-73150-data1.zip › 2022 Fanfone et al. source data WB panels/FigS2F source data ANNOTATED.pdf]

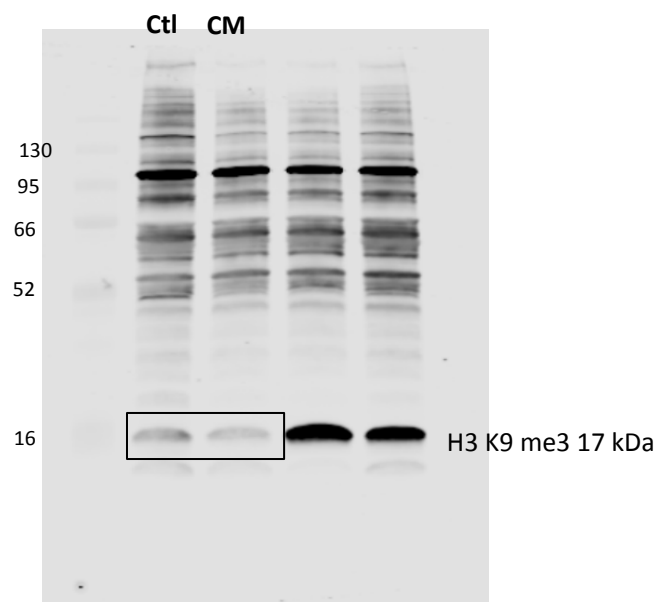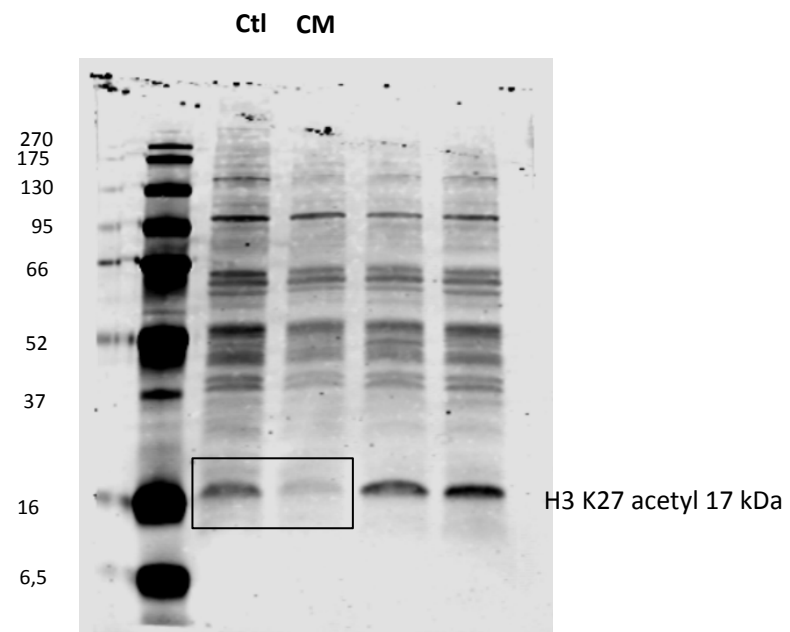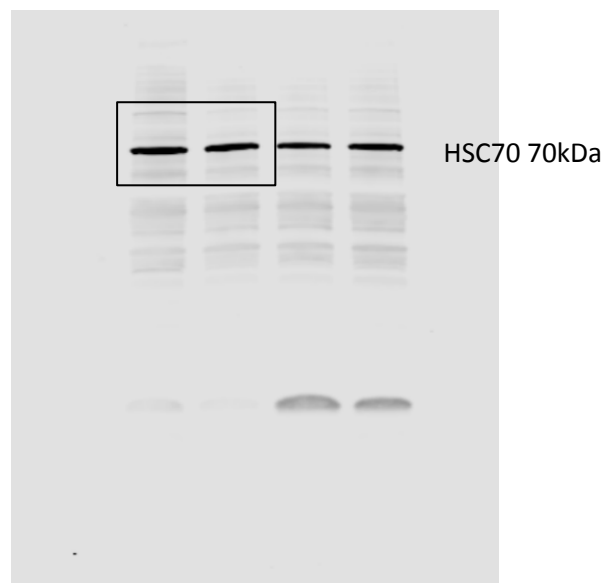

Supplementary Fig. 3C

Supplement: Source data 1. [file elife-73150-data1.zip › 2022 Fanfone et al. source data WB panels/FigS3C source data ANNOTATED.pdf]

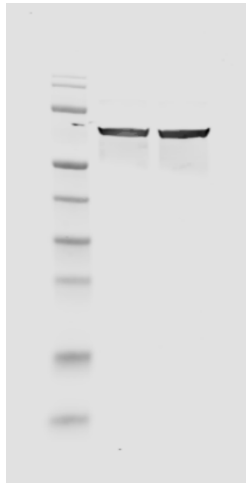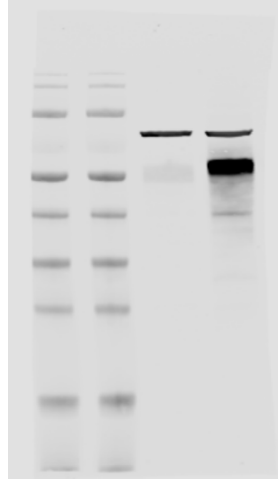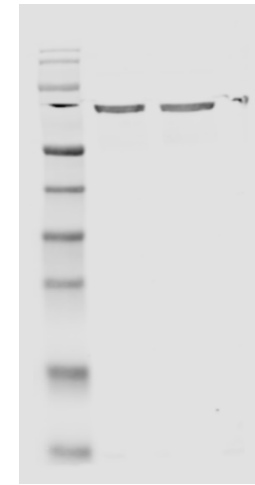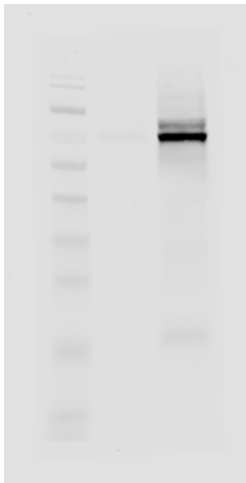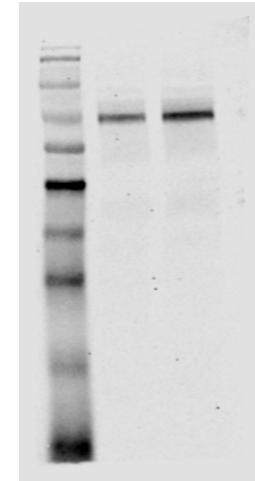

Figure 2B

Supplement: Source data 1. [file elife-73150-data1.zip › 2022 Fanfone et al. source data WB panels/Fig2B source data RAW.pdf]

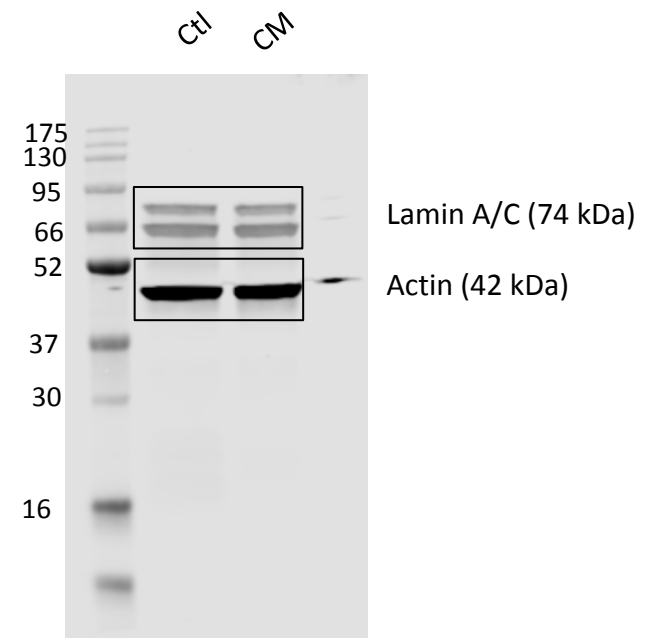

Supplementary Fig. 3K

Supplement: Source data 1. [file elife-73150-data1.zip › 2022 Fanfone et al. source data WB panels/FigS3K source data ANNOTATED.pdf]

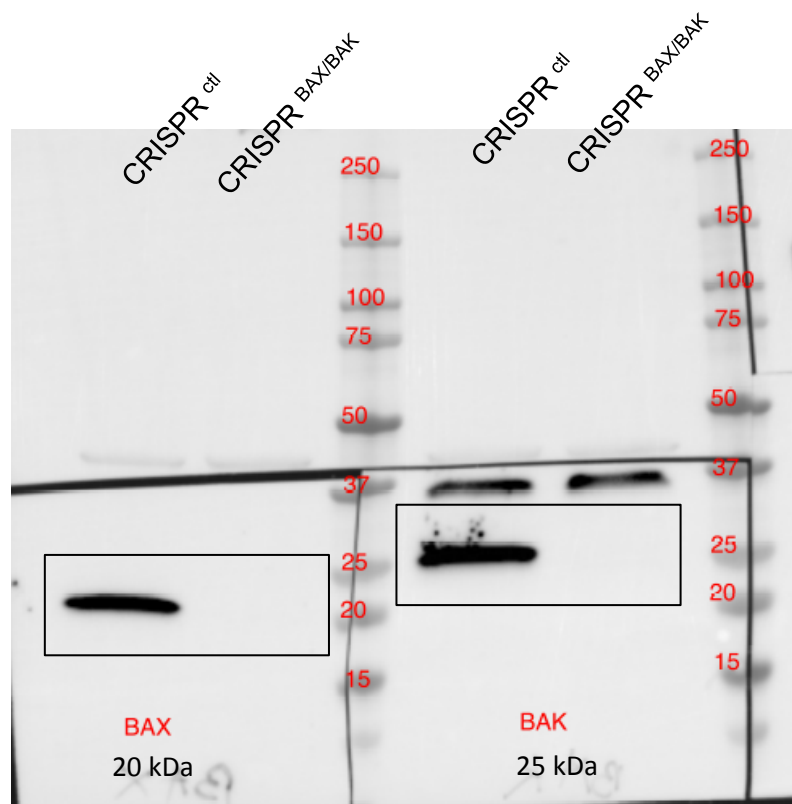

MDA-MB-231

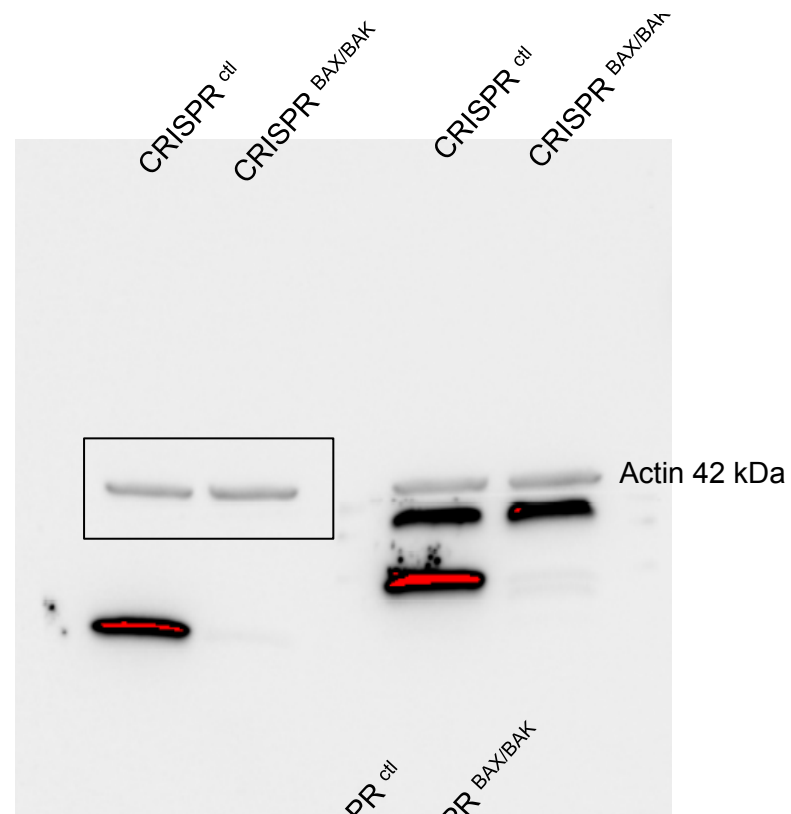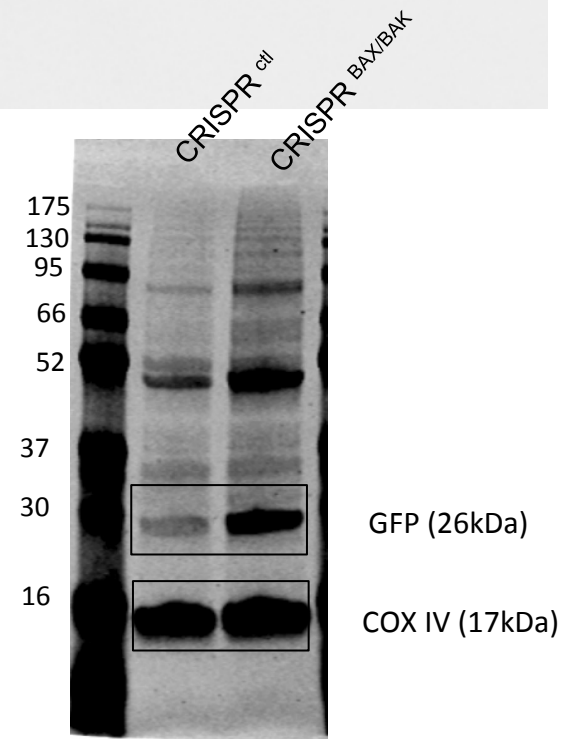

Supplementary Figure 1B

Supplement: Source data 1. [file elife-73150-data1.zip › 2022 Fanfone et al. source data WB panels/FigS1B source data ANNOTATED.pdf]

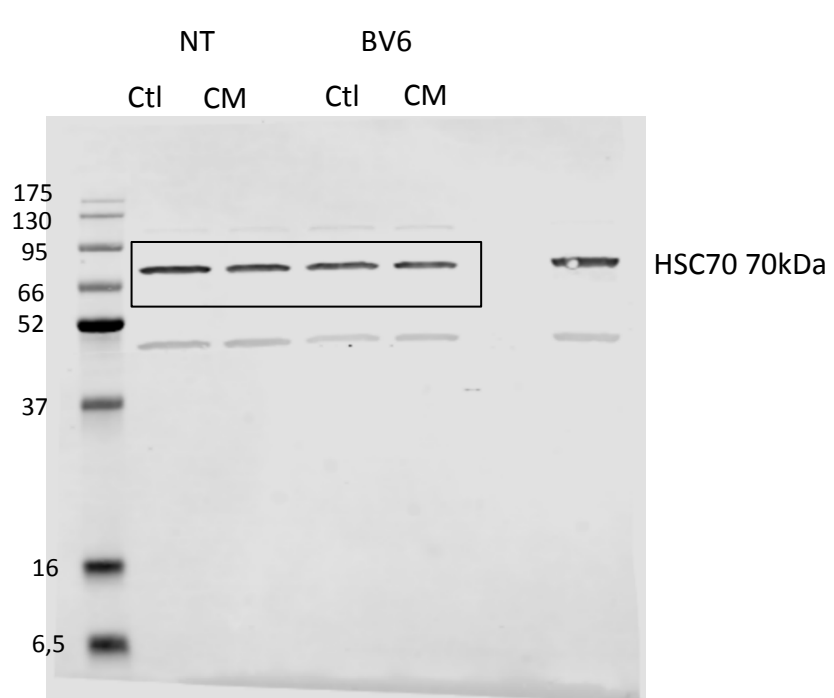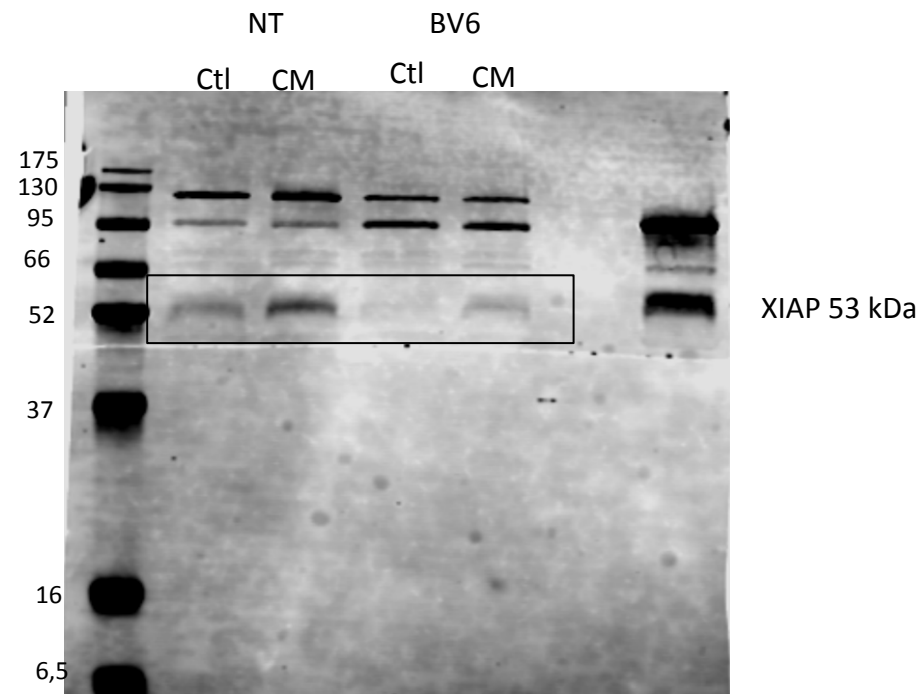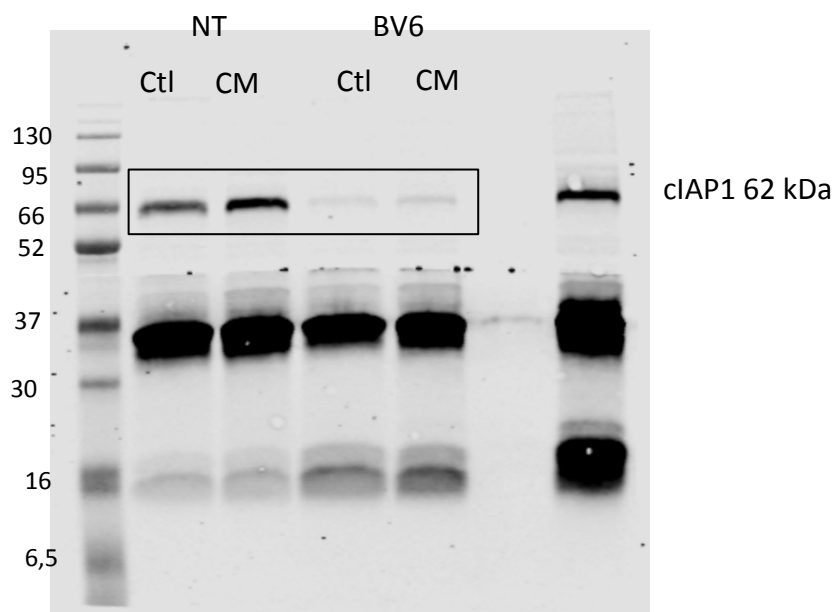

Figure 2J

Supplement: Source data 1. [file elife-73150-data1.zip › 2022 Fanfone et al. source data WB panels/Fig2J source data ANNOTATED.pdf]

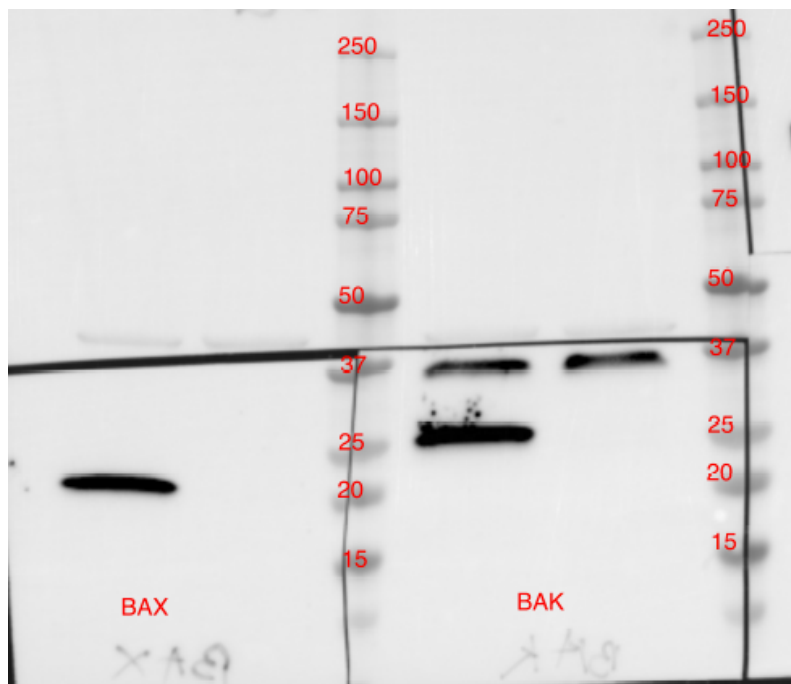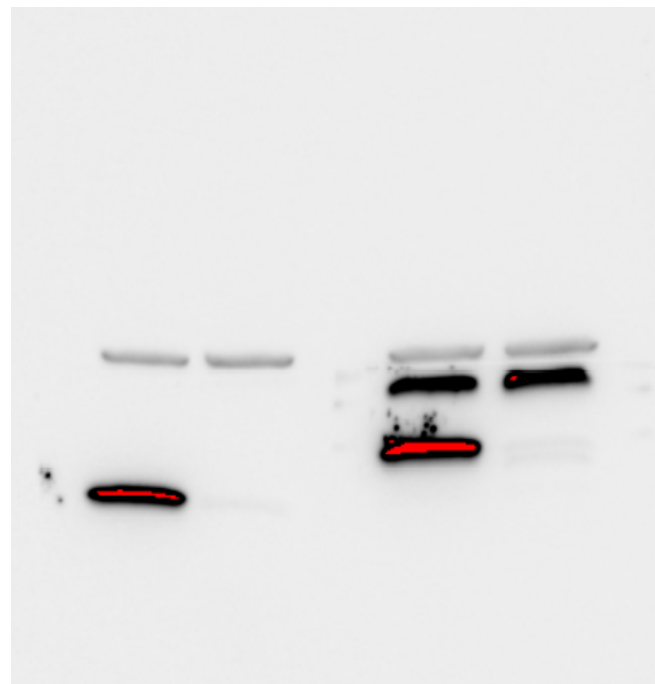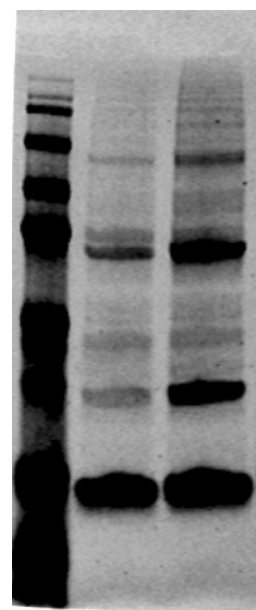

Supplementary Figure 1B

Supplement: Source data 1. [file elife-73150-data1.zip › 2022 Fanfone et al. source data WB panels/FigS1B source data RAW.pdf]

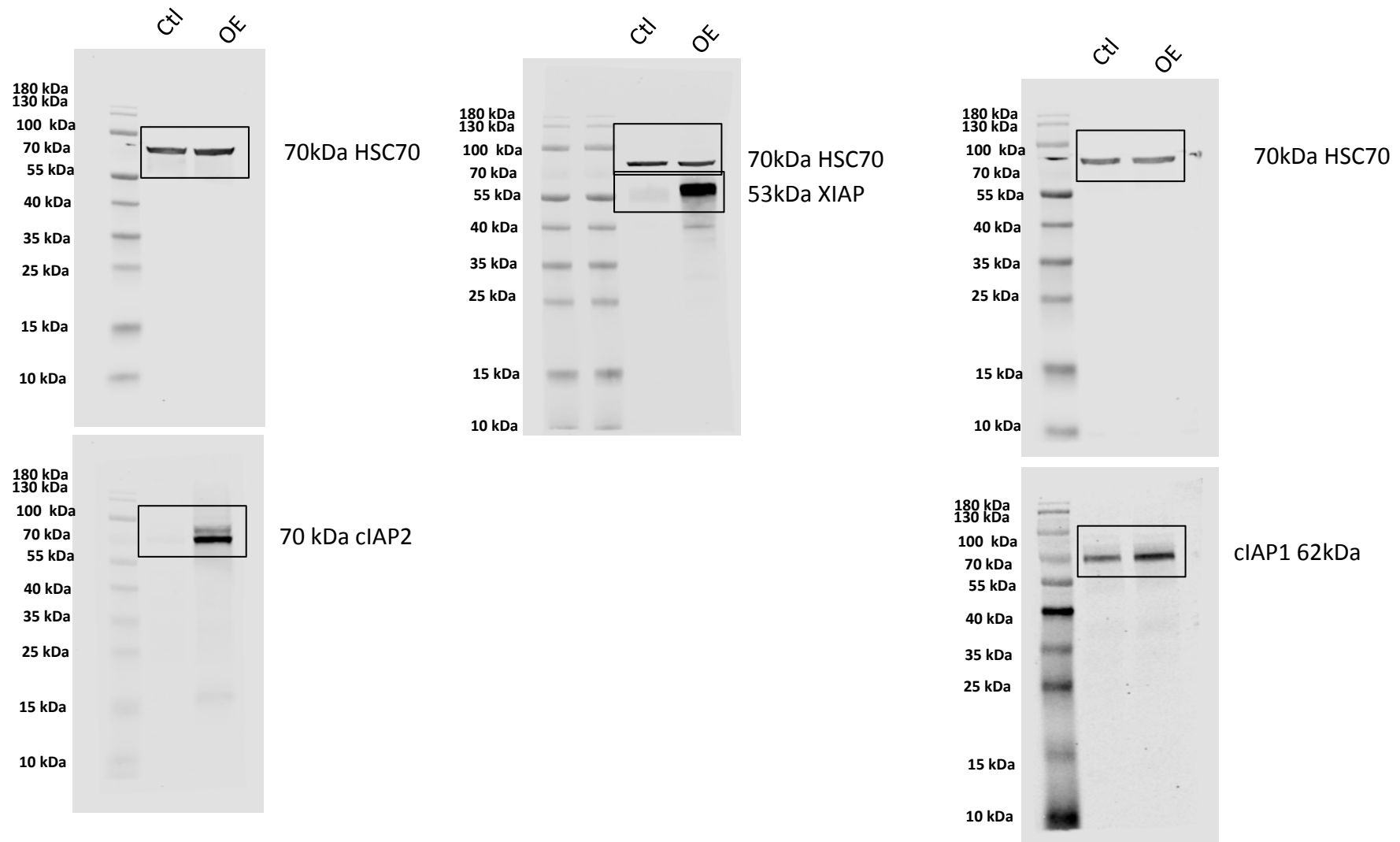

Figure 2B

Supplement: Source data 1. [file elife-73150-data1.zip › 2022 Fanfone et al. source data WB panels/Fig2B source data ANNOTATED.pdf]

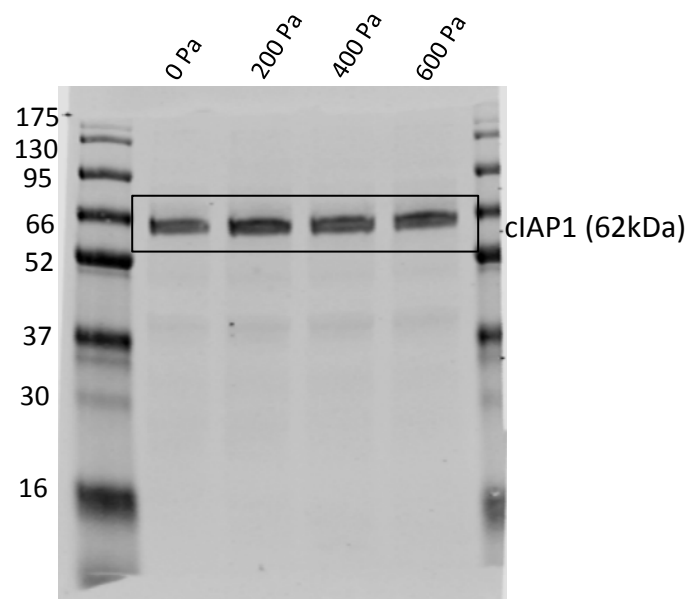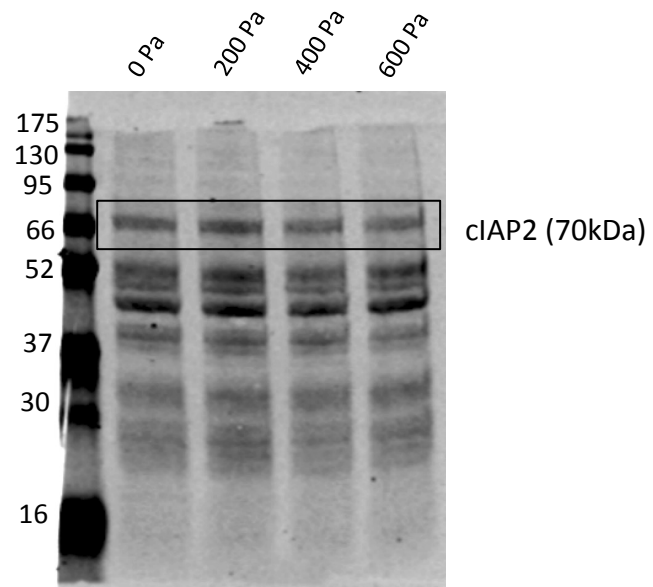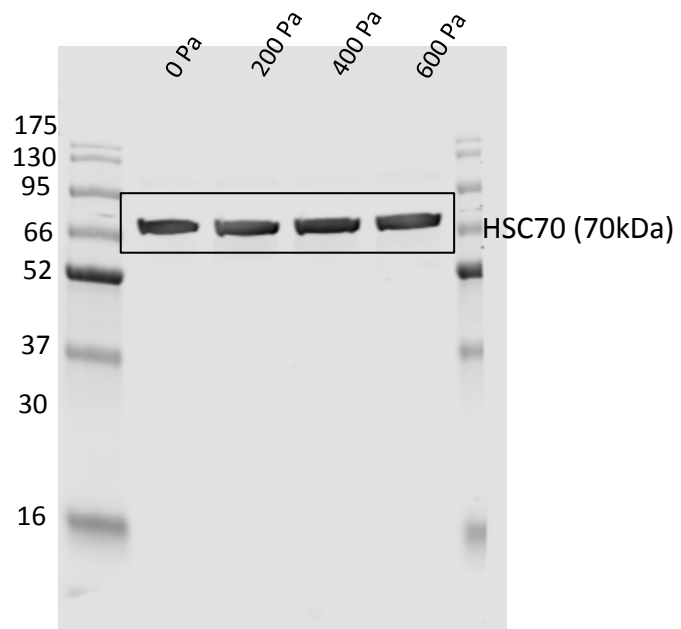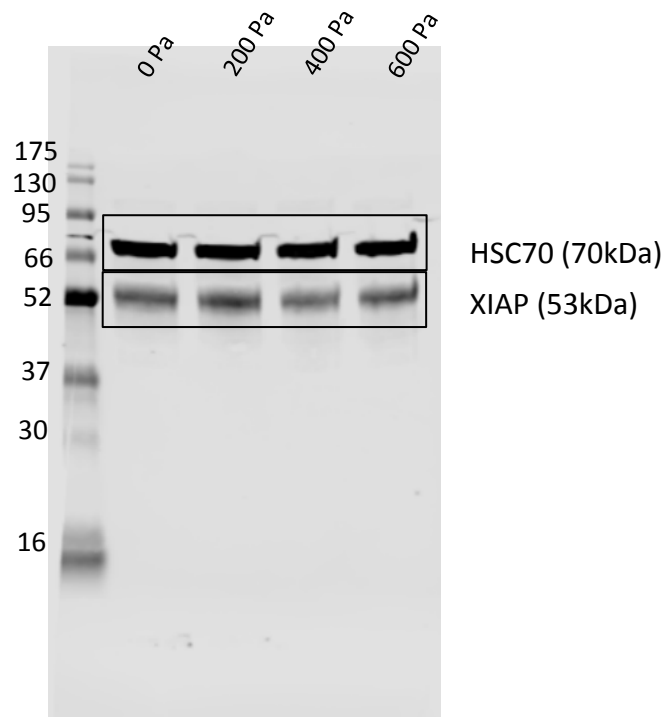

Supplementary Fig. 2A

Supplement: Source data 1. [file elife-73150-data1.zip › 2022 Fanfone et al. source data WB panels/FigS2A source data ANNOTATED.pdf]

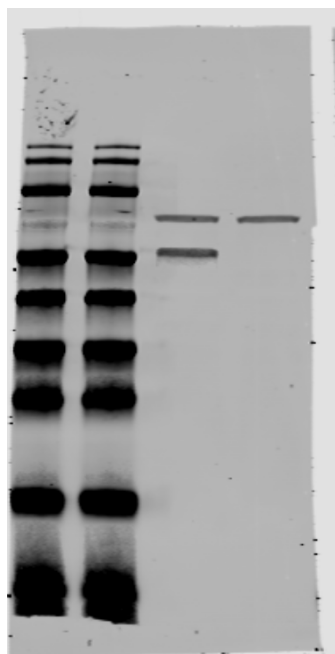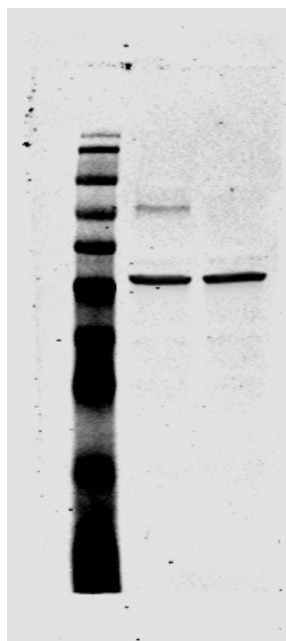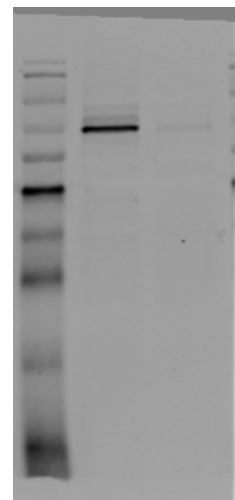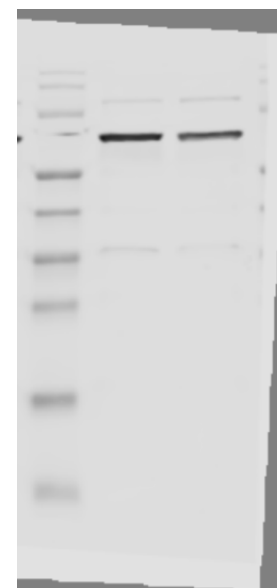

Figure 2F

Supplement: Source data 1. [file elife-73150-data1.zip › 2022 Fanfone et al. source data WB panels/Fig2F source data RAW.pdf]

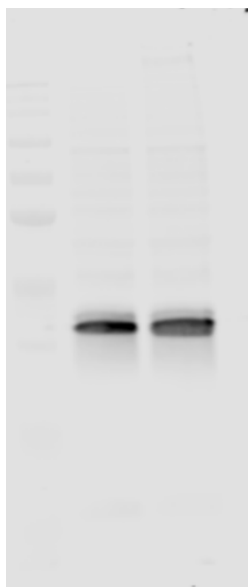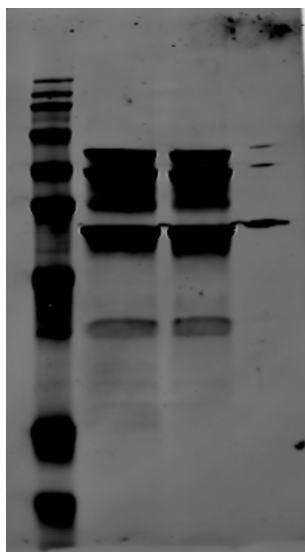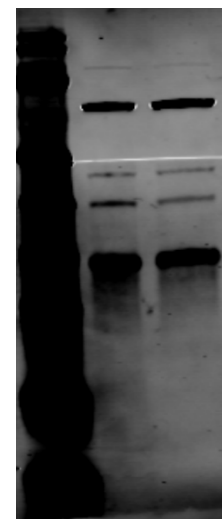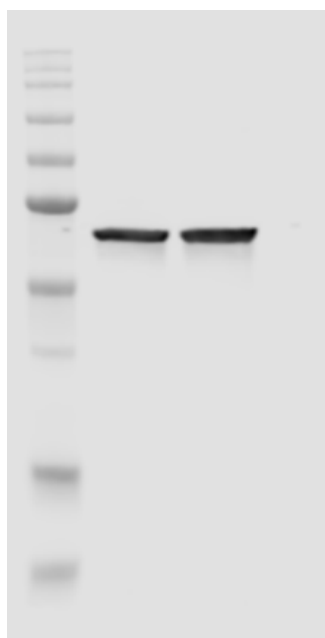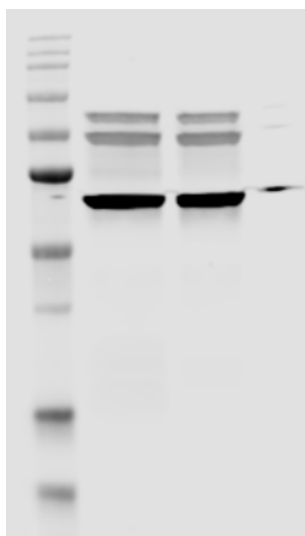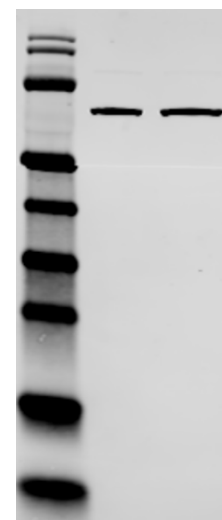

Supplementary Figure 1P

Supplement: Source data 1. [file elife-73150-data1.zip › 2022 Fanfone et al. source data WB panels/FigS1P source data RAW.pdf]

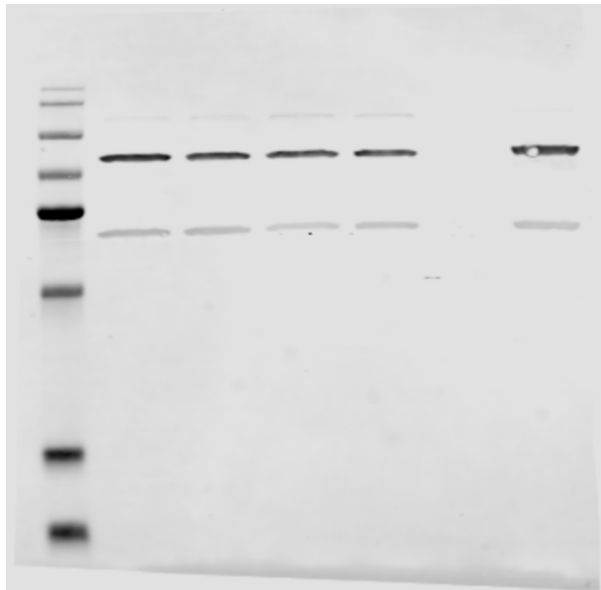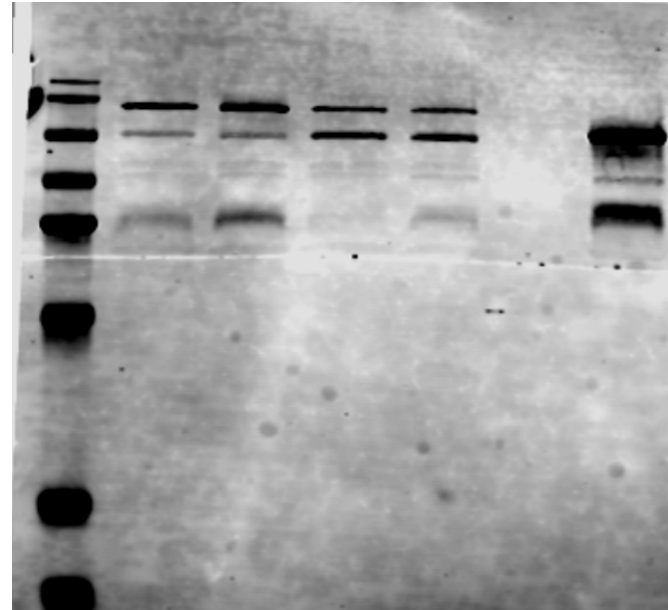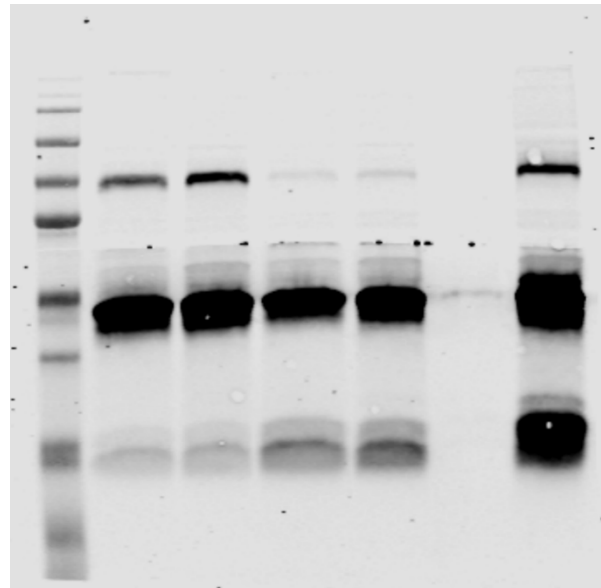

Figure 2J

Supplement: Source data 1. [file elife-73150-data1.zip › 2022 Fanfone et al. source data WB panels/Fig2J source data RAW.pdf]

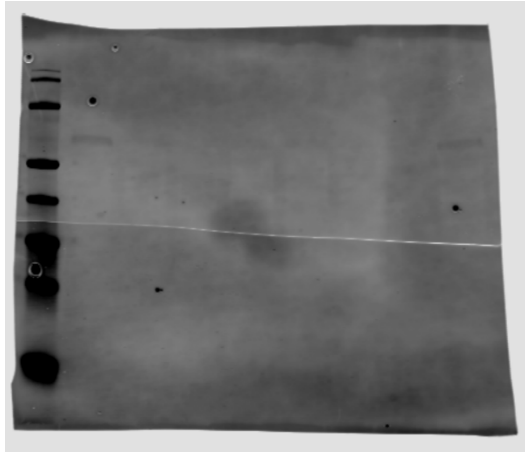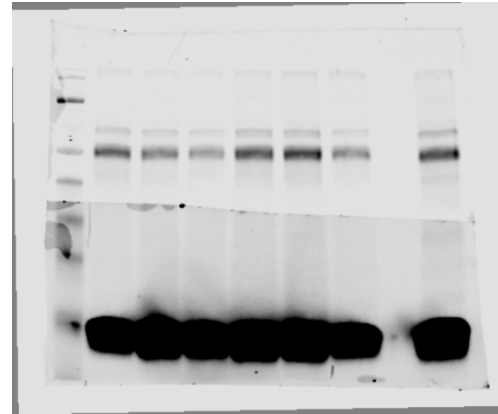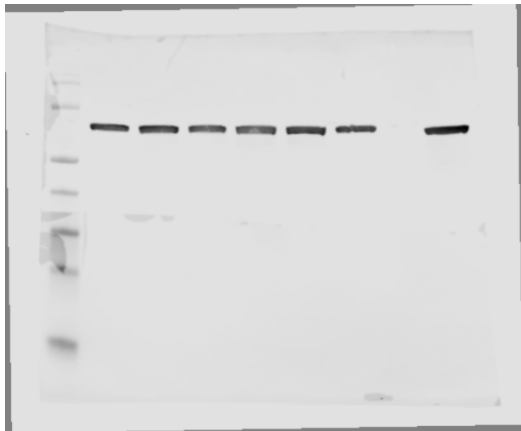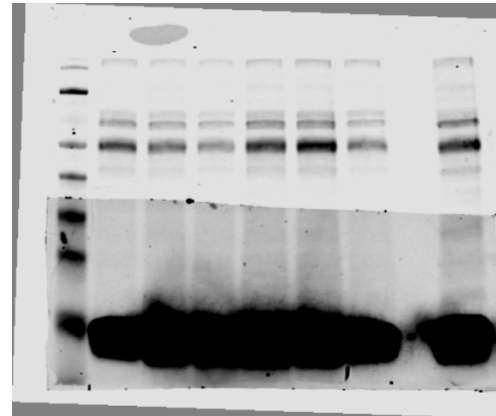

Figure 2H

Supplement: Source data 1. [file elife-73150-data1.zip › 2022 Fanfone et al. source data WB panels/Fig2H source data RAW.pdf]

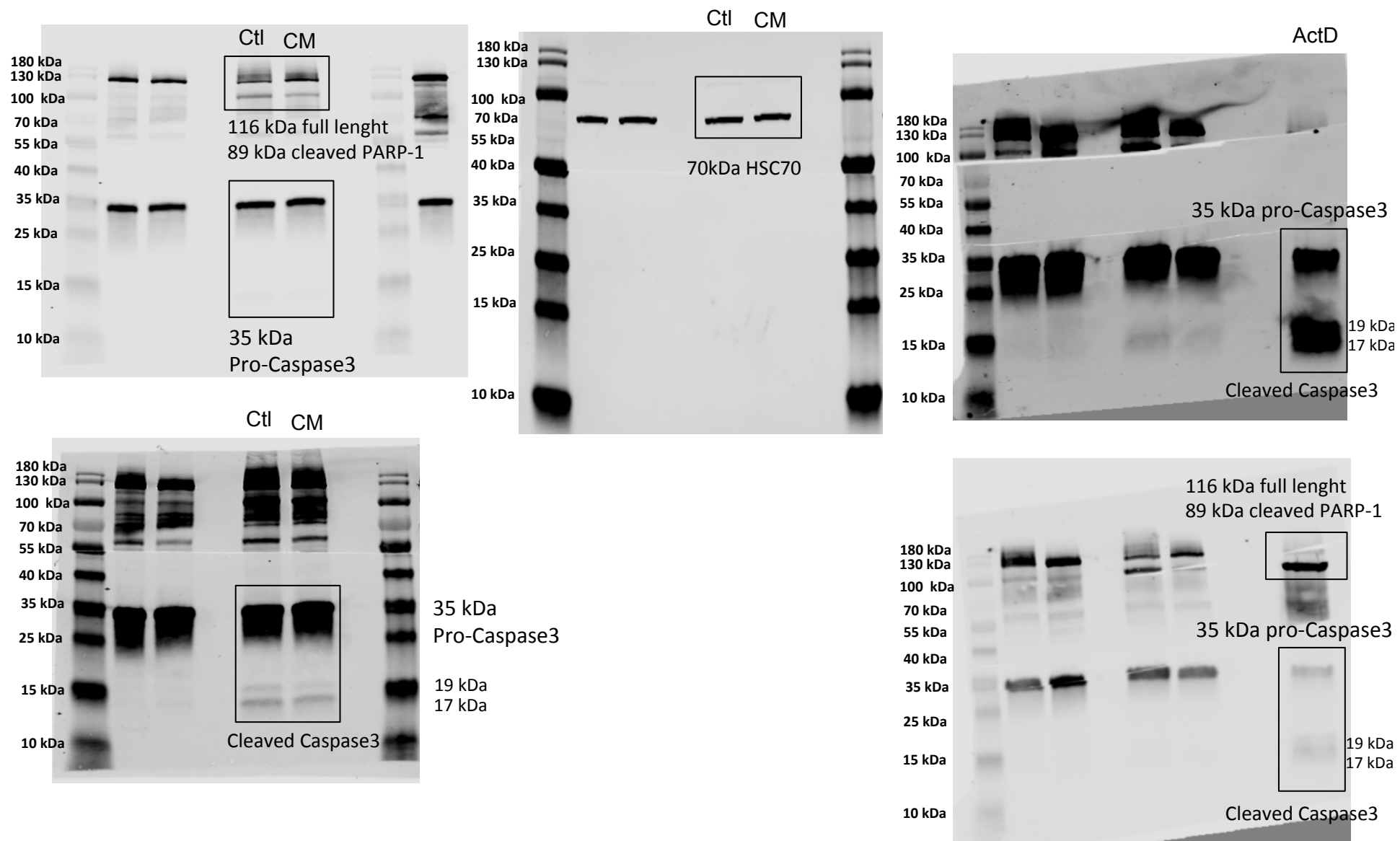

CRISPR Ctl MDA-MB-231

Figure 1K

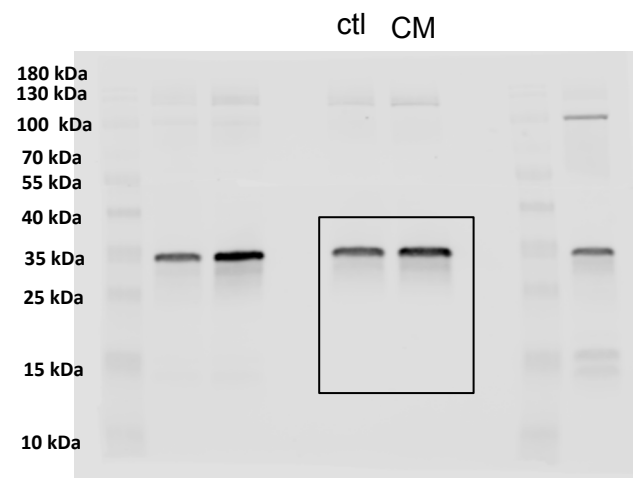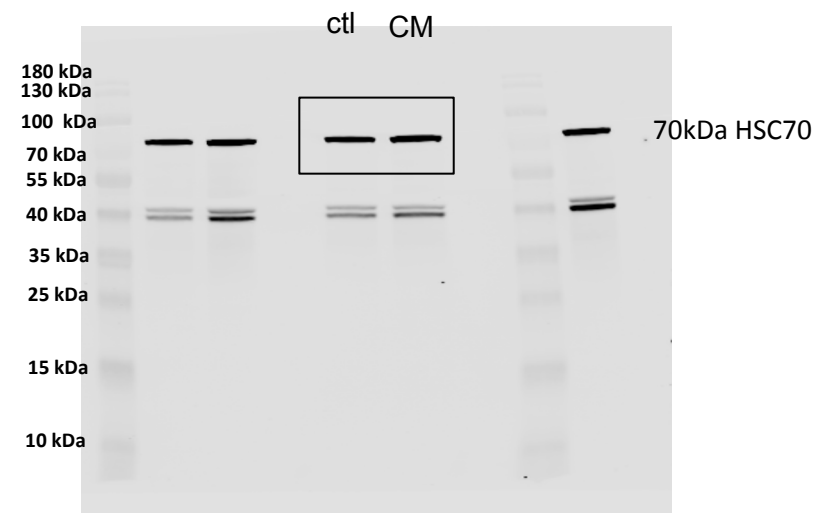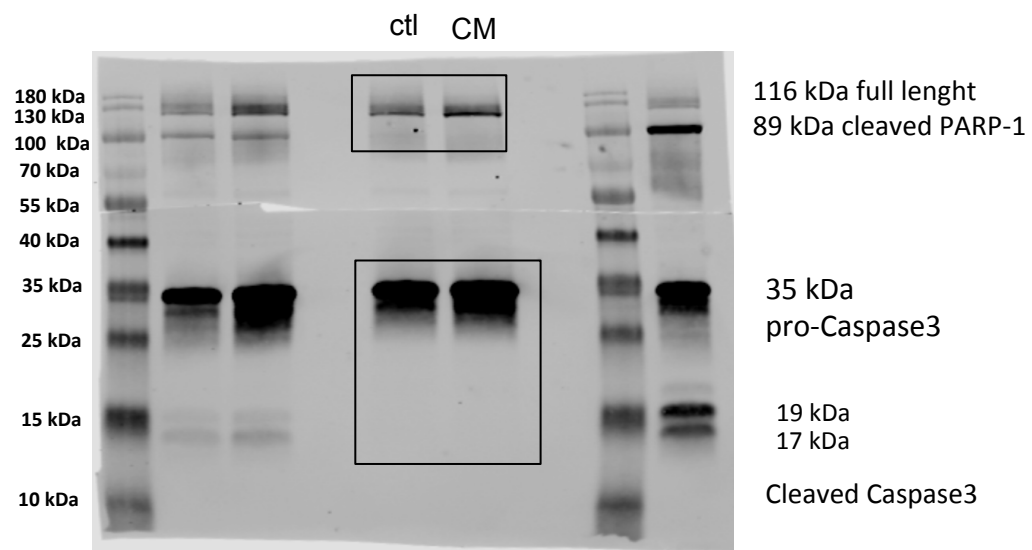

Figure 1K

CRISPR <sup>BAX/BAK</sup> MDA-MB-231

Supplement: Source data 1. [file elife-73150-data1.zip › 2022 Fanfone et al. source data WB panels/Fig1k source data ANNOTATED.pdf]
